# Recursive genome engineering decodes the evolutionary origin of an essential thymidylate kinase activity in *Pseudomonas putida* KT2440

**DOI:** 10.1128/mbio.01081-23

**Published:** 2023-09-21

**Authors:** Nicolas T. Wirth, Katja Rohr, Antoine Danchin, Pablo I. Nikel

**Affiliations:** 1 The Novo Nordisk Foundation Center for Biosustainability, Technical University of Denmark, Kongens, Lyngby, Denmark; 2 School of Biomedical Sciences, Li Ka Shing Faculty of Medicine, University of Hong Kong, Pokfulam, Hong Kong; Corporación CorpoGen, Bogotá D.C., Colombia

**Keywords:** *Pseudomonas putida*, thymidylate kinase, prophage, bacterial evolution, genome reduction, horizontal gene transfer, genome engineering

## Abstract

Thymidylate kinases (TMPKs) play an essential role in DNA biosynthesis across all domains of life by catalyzing dTMP phosphorylation to dTDP. In *Pseudomonas putida* KT2440, a model Gram-negative soil bacterium, *tmk* is disrupted by a 65-kb genomic island (GI), posing questions about the origin of the essential TMPK function. To solve this long-standing evolutionary riddle, we addressed three competing hypotheses: (i) assembly of two Tmk segments into a functional protein, (ii) complementation by a deoxynucleotide monophosphate kinase encoded within the GI, or (iii) fulfillment of the essential function by the product of *PP_3363*, yet another gene annotated as “thymidylate kinase.” Systematic genome engineering, quantitative physiology and targeted proteomics, complementation assays, phylogenetic analysis, and structure homology modeling were combined to investigate the role of genes within the GI. Our findings revealed that the GI-encoded dNMPK gene *PP_1964* plays a critical role in complementing the disrupted TMPK function—exposing a non-essential character for the native *PP_3363* gene and the *tmk* pseudogene. This dNMPK was found to be structurally related to that of bacteriophage T4, as part of a distinct evolutionary domain connected to mobile genetic elements and phages. The recursive genome reduction approach in this work deepens our understanding of the genetic architecture of a model bacterium while it provides evidence that the essential TMPK function has been acquired by horizontal gene transfer. Furthermore, the insights gained in the present study have broader implications for understanding the essentiality and functionality of dNMPK homologs in other bacteria.

**IMPORTANCE:**

Investigating fundamental aspects of metabolism is vital for advancing our understanding of the diverse biochemical capabilities and biotechnological applications of bacteria. The origin of the essential thymidylate kinase function in the model bacterium *Pseudomonas putida* KT2440, seemingly interrupted due to the presence of a large genomic island that disrupts the cognate gene, eluded a satisfactory explanation thus far. This is a first-case example of an essential metabolic function, likely acquired by horizontal gene transfer, which “landed” in a locus encoding the same activity. As such, foreign DNA encoding an essential dNMPK could immediately adjust to the recipient host—instead of long-term accommodation and adaptation. Understanding how these functions evolve is a major biological question, and the work presented here is a decisive step toward this direction. Furthermore, identifying essential and accessory genes facilitates removing those deemed irrelevant in industrial settings—yielding genome-reduced cell factories with enhanced properties and genetic stability.

## INTRODUCTION

*Pseudomonas putida* KT2440 is a Gram-negative, soil-dwelling bacterium that became a model bacterial *chassis* for both fundamental research and synthetic biology due to its versatile metabolism, stress resistance and robustness under harsh operating conditions (1 – 3). Among many other applications, both wild-type and engineered strains of *P. putida* have been implemented for xenobiotic biodegradation (4), biopolymer and biofuel production (5) and even new-to-nature biocatalysis (6). However, despite the wealth of knowledge amassed on the metabolic pathways and physiological properties of *P. putida*, significant gaps remain in our understanding of some essential genes and their functions. A bacterial *chassis* for synthetic biology is made up of a core set of genes that are not susceptible to voluntary alteration, together with specific genes that encode a number of functions of interest (e.g., physiological traits relevant for industrial applications) (7 – 9). Thymidylate kinase (TMPK; EC 2.7.4.9) belongs to the conserved set of genes in any given bacterial *chassis* (10 – 12). However, the gene encoding this function in *P. putida* KT2440 is interrupted by a DNA insert of foreign origin, and the biochemical process that allows the cell to perform the essential TMPK function is yet to be identified—an evolutionary riddle that has eluded a convincing explanation in spite of decades of research in *P. putida* metabolism.

**Fig 1:**
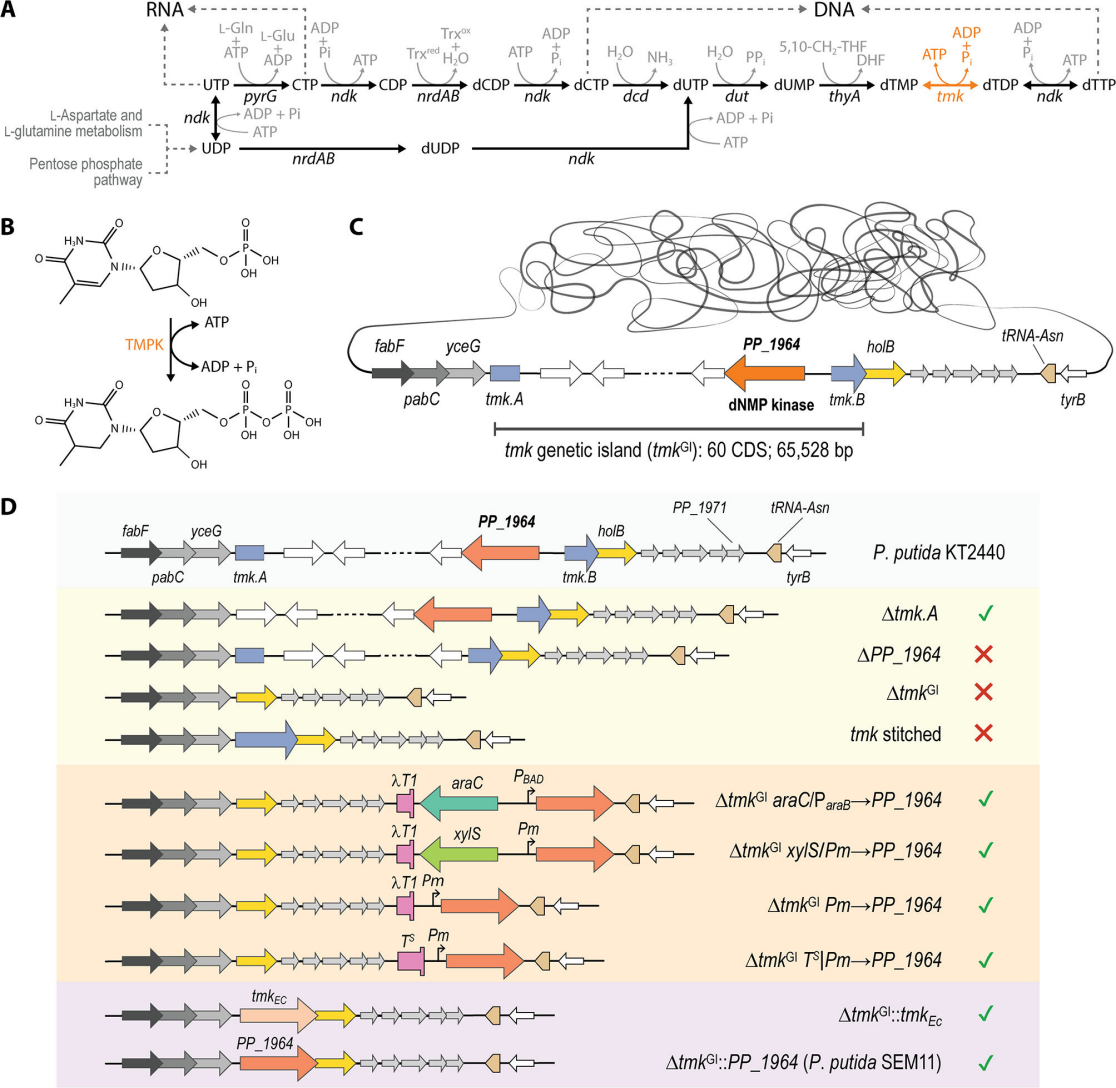
Biochemistry and genetics underlying *de novo* biosynthesis of pyrimidine deoxyribonucleotides in *Pseudomonas putida* KT2440. (**A**) Overview of the biochemical pathways involved in the formation of CTP and UTP (i.e., RNA building blocks) as well as dCTP and dTTP (i.e., DNA components). Genes encoding key enzymes are indicated alongside the relevant reaction, together with the relevant cofactors. (**B**) Biochemical reaction catalyzed by thymidine 5′-monophosphate kinase (TMPK). (**C**) Chromosomal locus bearing the TMPK-encoding gene *tmk* in *P. putida* KT2440. This gene is disrupted by a genetic island (*tmk* ^GI^) composed of 60 coding sequences (CDS). (**D**) Genetic designs implemented in this study to explore the (potential) essential nature of *tmk* and *PP_1964* (annotated as a dGMP/dTMP/dihydroxy-CMP kinase). The ✔ and × symbols indicate success or failure, respectively, in the implementation of each of the depicted genetic architectures. Abbreviations are as follows: *dcd*, deoxycytidine deaminase; *dut*, dUTP diphosphatase (i.e., dUTPase); *fabF*, enoyl-coenzyme A (CoA) hydratase; *holB*, subunit α of the DNA polymerase III; *pabC*, *p*-aminobenzoate synthase; *tyrB*, tyrosine synthase; *ndk*, nucleoside diphosphate kinase (i.e., NDPK); *nrdA*, subunit α of the ribonucleoside-diphosphate reductase; *nrdB*, subunit β of the ribonucleoside-diphosphate reductase; *pyrG*, CTP synthase (i.e., CTPS); *thyA*, thymidylate synthase; *tmk*, thymidine 5′-monophosphate kinase (i.e., TMPK); *tmk_Ec_*, *tmk* gene from *Escherichia coli* MG1655; SEM11, *P. putida* strain (derived from reduced-genome *P. putida* SEM10) carrying a Δ*tmk* ^GI^::dNMPK*_Pp_* element in the chromosome.

The synthesis of deoxynucleoside diphosphates represents the limiting step of DNA biosynthesis (Fig. 1A), with TMPK playing the essential role of catalyzing the ATP-dependent phosphorylation of deoxythymidine 5′-monophosphate (dTMP) to deoxythymidine 5′-diphosphate (dTDP; Fig. 1B). The substrate of TMPK, dTMP, originates either from the thymidine kinase (TK; EC 2.7.1.21)-dependent salvage pathway or the thymidylate synthase (TS; EC 2.1.1.45)-mediated *de novo* biosynthesis pathway. TMPK belongs to the NMP kinase family, characterized by a highly conserved fold across all three domains of life (13)—but displaying a less conserved amino acid sequence (14). These enzymes possess three crucial segments: the LID domain, the NMP binding domain and the CORE domain. The flexible LID domain partially encloses the phosphate donor to facilitate phosphate transfer, while the CORE domain encompasses the phosphate binding loop (P-loop) and the ATP binding site. The P-loop in the NMP domain contains structural elements vital for substrate recognition and catalysis, binding and positioning the α and β phosphoryl groups of ATP (15). The essentiality of these biochemical reactions (and, in particular, the TMPK-mediated formation of dTDP) has been explored and validated in several organisms, including *Escherichia coli* (16), *Saccharomyces cerevisiae* (17) and human cells (18).

The KEGG database fails to identify a TMPK activity in *P. putida* KT2440 (19), in line with the observations reported when the genome sequence of this bacterium was re-annotated (20)—such metabolic scenario is, however, extremely unlikely considering the enzyme essentiality. The canonical *tmk* gene, present in other *Pseudomonas* strains as an intact coding sequence (CDS), is disrupted by a 65-kb genomic island (GI) in *P. putida* KT2440 (21) and its parental strain, *P. putida* mt-2. This GI, which structurally resembles a prophage (22), is one of the largest found in the chromosome of this strain, splitting the *tmk* gene into two segments of 294 bp and 339 bp, respectively (Fig. 1C). The integration and potential inactivation of *tmk* without any apparent detrimental effect on the organism’s prototrophy is all the more intriguing in light of the fact that the downstream end of the gene overlaps with the *holB* gene. HolB is the δ′-subunit of DNA polymerase III, which plays a major role in DNA synthesis during chromosomal replication (23, 24). The inserted GI contains an arsenate resistance operon and a cluster of biosynthesis genes (25, 26), flanked by a site-specific recombinase (encoded by *PP_1962*) with strong homology to the XerC integrase of bacteriophage λ (27). Such a genome structure is absent in all five closely related *P. putida* strains that have been sequenced (28) and further contains three additional mobile genetic elements (i.e., PP_5490; a site-specific DNA recombinase of the Tn*3* family; PP_1931, an IS*1182*-like element of the IS*Ppu*16 family transposase; and PP_1960, a site-specific integrase)—which may indicate horizontal gene transfer. The very last gene in the GI of strain KT2440, *PP_1964*, is predicted to encode a deoxynucleotide monophosphate kinase (dGMP/dTMP/dihydroxy-CMP kinase or, in short, dNMPK*_Pp_*).

Three hypotheses could explain the lack of an identified TPMK in *P. putida* KT2440. In particular, (i) the two *tmk* gene segments could be expressed separately and assembled into a functional TMPK protein, (ii) the GI-encoded dNMPK*_Pp_* complements the TMPK function, or (iii) another gene within the *P. putida* KT2440 genome (i.e., outside the *tmk* locus) fulfills this essential reaction. Here, we sought to elucidate the essentiality and functional roles of genes within the GI flanked by the two *tmk* moieties in *P. putida* KT2440. Combining recursive chromosomal modifications (via stitching, re-shuffling, and systematic gene deletions and insertions) with complementation experiments, quantitative physiology, proteomics, phylogenetic analysis and homology modeling, our work resolves the long-standing question of the critical dNMPK*_Pp_* role and the GI wherein the gene lies. By deepening our understanding of the genetic architecture and metabolic capabilities of model environmental bacteria, we are paving the way for further exploitation of *P. putida* both in fundamental and applied research.

## RESULTS

### Systematic genome engineering of *P. putida* KT2440 identifies *PP_1964*, and not *tmk*, as essential for nucleotide metabolism

In order to explore the three hypotheses advanced to explain the origin and functionality of dNMPK*_Pp_*, we adopted a synthetic biology approach whereby a series of genetic constructs were designed and tested in *P. putida* KT2440 (Fig. 1D; Table 1). The gene annotated as to encode an alternative thymidylate kinase, *PP_3363* (20), was chosen as a first obvious target. This CDS had not been deemed essential in the high-quality genome-scale metabolic reconstruction of *P. putida* KT2440 (29) and nor had it been identified as necessary for citrate-dependent growth of this strain (30). Additionally, we could not find experimental validation of its predicted function for any of the 159 homologs identified via BLAST search (details not shown). The amino acid sequence of PP_3363 shares a 22.4% sequence identity with TMPK of *P. putida* F1, one of the sequenced strains closest to *P. putida* KT2440 (21). Moreover, this protein has a maximum identity of 22% with bacterial TMPKs whose structure has been fully solved—e.g., 5TMP from *E. coli* (17.24%) and 4EDH from *P. aeruginosa* (20.5%). In contrast, *P. putida* F1 TMPK shares an 80.5% identity with *P. aeruginosa* PAO1 TMPK and a 50.5% identity with *E. coli* MG1655 TMPK. It was thus not surprising that *PP_3363* could be deleted without any observable growth defects (Table 1), indicating that this supposed “thymidylate kinase” is not essential in *P. putida* KT2440.

**TABLE 1:** Bacterial strains and plasmids used in this study

Strain	Relevant characteristics^*a*^	Reference or source
*Escherichia coli*		
DH5α λ*pir*	Cloning host; F^–^ λ^–^ *endA1 glnX44*(AS) *thiE1 recA1 relA1 spoT1 gyrA96*(Nal^R^) *rfbC1 deoR nupG* Φ80(*lacZ*Δ*M15*) Δ(*argF-lac*)*U169 hsdR17*(*r_K_* ^–^ *m_K_^+^*), λ*pir* lysogen	(31)
MG1655	Wild-type strain; F^–^ λ^–^ *ilvG rfb-50 rph-1*	(32)
JHR114	Derivative of strain MG1655, Δ*tmk araC/P_BAD_* →*tmk_Mt_*; Sm^R^	(33)
*Pseudomonas putida*		
KT2440	Derivative of *P*. *putida* mt-2 (34) cured of the TOL plasmid pWW0	(35)
EM42	Derivative of strain KT2440; Δprophage1 Δprophage4 Δprophage3 Δprophage2 ΔTn*7*Δ*endA-1* Δ*endA-2* Δ*hsdRMS* Δflagellum ΔTn*4652*	(36)
SEM10	Derivative of strain EM42; Δ*PP_0052* Δ*PP_0772* Δ*PP_1239* Δ*PP_1775* Δ*PP_1952* Δ*PP_2045* Δ*PP_2876* Δ*PP_3291* Δ*benABCD* Δ*pvdD*	(37)
Δ*PP_3363*	Derivative of strain KT2440; Δ*PP_3363*	This work
NTW1804	Derivative of strain KT2440; Δ*tmk* ^GI^::*tmk_Ec_*	This work
NTW1797	Derivative of strain KT2440; Δ*tmk* ^GI^ *xylS*/*Pm*→*PP_1964*	This work
NTW1788	Derivative of strain KT2440; Δ*tmk* ^GI^ *araC*/*P_araB_* →*PP_1964*	This work
NTW1835	Derivative of strain KT2440; Δ*tmk* ^GI^ λT1|*Pm*→*PP_1964*	This work
NTW1834	Derivative of strain KT2440; Δ*tmk* ^GI^ *T^S^* |*Pm*→*PP_1964*	This work
NTW1283	Derivative of strain SEM10; Δ*tmk.A*	This work
NTW1282	Derivative of strain SEM10; Δ*tmk* ^GI^::*tmk_Ec_*	This work
SEM11	Derivative of strain SEM10; Δ*tmk* ^GI^::*PP_1964*	This work
Plasmids		
pGNW2	Suicide vector used for genetic manipulations in Gram-negative bacteria; *oriT, traJ, lacZα, ori*(R6K)*,* P*_14g_(BCD2)→msfGFP*; Km^R^	(38)
pSNW2	Derivative of vector pGNW2; with the *msfGFP* translation initiation sequence replaced by the strong *BCD2* translational coupler	(37)
pQURE6·H	Conditionally-replicating vector; XylS/*Pm*→*I-SceI* and *P_14g_(BCD2*)→*mRFP*; Gm^R^	(37)
pSNW2∙Δ*tmk.A*	Derivative of vector pSNW2 carrying HAs to delete the *tmk* part upstream of the GI in *P. putida* KT2440	This work
pSNW2∙Δ*PP_1964*	Derivative of vector pSNW2 carrying HAs to delete *PP_1964*	This work
pSNW2∙Δ*tmk* ^GI^	Derivative of vector pSNW2 carrying HAs to delete the entire GI including *tmk* that it disrupted	This work
pSNW2∙stitch-*tmk*	Derivative of vector pSNW2 carrying HAs to delete the entire GI, restoring the original *tmk* sequence	This work
pSNW2∙*Pm*→*PP_1964*	Derivative of vector pSNW2 carrying *Pm*→*PP_1964* flanked by HAs for integration between *PP_1971* and *PP_t63*	This work
pSNW2∙*xylS*/*Pm*→*PP_1964*	Derivative of vector pSNW2 carrying *xylS*/*Pm*→*PP_1964* flanked by HAs for integration between *PP_1971* and *PP_t63*	This work
pSNW2∙*araC*/ *P_araB_* →*PP_1964*	Derivative of vector pSNW2 carrying *araC*/*P_araB_* →*PP_1964* flanked by HAs for integration between *PP_1971* and *PP_t63*	This work
pSNW2∙*Pm*→*PP_1964*	Derivative of vector pSNW2 carrying *Pm*→*PP_1964,* flanked by HAs for integration between *PP_1971* and *PP_t63*	This work
pSNW2∙*T^S^* |*Pm*→*PP_1964*	Derivative of vector pSNW2 carrying the strong transcriptional terminator T^S^ (39) isolating *Pm*→*PP_1964,* flanked by HAs for integration between *PP_1971* and *PP_t63*	This work
pSNW2∙Δ*tmk* ^GI^::*tmk_Ec_*	Derivative of vector pSNW2 carrying HAs to replace the entire GI including *tmk* that it disrupted with *tmk_Ec_*	This work
pSNW2∙Δ*tmk* ^GI^:: *PP_1964*	Derivative of vector pSNW2 carrying HAs to replace the entire GI including *tmk* that it disrupted with *tmk_Ec_*	This work
pMBEC6∙*PP_1964*	Derivative of the multiplex base-editing plasmid pMBEC6 (40) harboring three synthetic gRNAs targeting *PP_1964*	This work
pEX128·gRNA	Plasmid carrying a template gRNA scaffold; Amp^R^	(40)
pRHA-CDC8	Derivative of vector pACYC184 (41); *rhaRS/P_rhaBAD_*, *oriV*(p15A), *CDC8* from *Saccharomyces cerevisiae*, *C*-terminal *FLAG* epitope; Cm^R^ Tc^R^	(33)
pRHA·*tmk_Ec_*	Derivative of vector pRHA-CDC8 containing *rhaRS/P_rhaBAD_* →*tmk_Ec_*, Cm^R^ Tc^R^	(33)
pRHA·*tmk_Pa_*	Derivative of vector pRHA-CDC8 containing *rhaRS/P_rhaBAD_* →*tmk_Pa_*, Cm^R^ Tc^R^	(33)
pRHA·*PP_1964*	Derivative of vector pRHA-CDC8 containing *rhaRS/P_rhaBAD_* →*PP_1964*, Cm^R^ Tc^R^	This work
pSEVA441	Standard cloning vector; *oriV*(pRO1600/ColE1); Sm^R^	(42, 43)
pSEVA4418	Derivative of vector pSEVA441; *rhaRS/P_rhaBAD_*, *oriV*(pRO1600/ColE1); Sm^R^	This work
pS4418∙*tmk_Ec_*	Derivative of vector pSEVA441 containing *rhaRS/P_rhaBAD_* →*tmk_Ec_* from plasmid pRHA-*tmk_Ec_* as cargo; Sm^R^	This work

^*a*^ Antibiotic markers: Amp, ampicillin; Cm, chloramphenicol; *Km*, kanamycin; *Gm*, gentamicin; *Sm*, streptomycin; and *Tc*, tetracyclin. *HA*, homology arms.

Next, we attempted to remove the entire GI comprising the prophage (*tmk* ^GI^) and the two flanking *tmk* halves (a design termed Δ*tmk* ^GI^, Fig. 1D) to expose a potential TMPK activity encoded outside this genome region. In this specific genetic design, the *holB* gene was left in the exact genome context where *tmk* was originally located—thereby using the regulatory features of *tmk*. However, we were unable to implement this genetic architecture in spite of multiple attempts, suggesting that the gene cluster harbors essential functions (and, most likely, a TMPK activity). We then aimed at recovering the intact *tmk* gene by stitching its two halves together and removing the intervening GI. The resulting CDS, cloned on the pSNW2 suicide vector to perform the stitching procedure (plasmid pSNW2∙stich-*tmk*; Table 1), has the same length as the *tmk* gene from other *Pseudomonas* species. Indeed, the resulting reconstituted *tmk* sequence should encode a protein displaying 99.5% amino acid sequence identity to TMPK from *P. putida* F1, with only a single amino acid substitution close to the *C*-terminus. This approach did not work despite numerous implementation efforts, including the use of CRISPR-Cas9 counter-selection (38), even though *P. putida* KT2440 *tmk* has >98% identity with orthologs of several other *Pseudomonas* species (e.g., *P. taiwanensis* VLB120, *P. putida* CFA, or *P. putida* DOT1E). Restoring the original CDS by stitching the two *tmk* fragments of strain KT2440 failed also when modifying the predicted translational coupling architecture with the *holB* gene to 5′-TG*ATG*-3′. In this modification, the *STOP* codon of *tmk*, highlighted in bold, should overlap with the *START* codon of *holB*, indicated in italics. To further investigate the essentiality of the split *tmk* CDS, we further performed a defined deletion of the upstream half (Δ*tmk.A*, Fig. 1D), which succeeded without any noticeable changes in growth phenotypes under several growth conditions (results not shown). In contrast, we were unable to remove the GI-encoded dNMPK*_Pp_* gene *PP_1964* (Δ*dNMPK*, Fig. 1D)—indicating that it might be essential.

Overall, these results indicate that the gene clusters within the GI of strain KT2440, which has a strong structural resemblance with a prophage, contain essential functions and further suggest that this activity may reside in dNMPK*_Pp_*. Therefore, to further test if the *PP_1964* CDS encodes a dTMPK function, we conducted a series of experiments that involved replacing the entire gene cluster with either a *tmk* gene from *E. coli* or the native *PP_1964*, the details of which are outlined in the next section.

### The entire GI can be excised from the chromosome of *P. putida* KT2440 via functional replacement with either *tmk* from *E. coli* or the endogenous *PP_1964* gene

Based on the experimental evidence described above, it appeared that dNMPK*_Pp_* plays an essential function encoded in the GI gene clusters of *P. putida*. To further substantiate this idea, the entire *tmk*-prophage gene cluster was replaced with the *tmk* gene from *E. coli* MG1655 (*tmk_Ec_*), yielding *P. putida* NTW1804 (Table 1). Such functional replacement (Δ*tmk* ^GI^::*tmk_Ec_*, Fig. 1D) succeeded readily, indicating that TMPK is indeed the essential function encoded within the viral GI region. In this strategy, *holB* was translationally coupled to the *tmk_Ec_* gene via the aforementioned genetic configuration (5′-TG*ATG*-5′). In a previous study by Páez-Espino et al. (28), deletion of the *ars1* operon, encoded within the *tmk* ^GI^ region, amplified the sensitivity of the mutant strain to arsenate (AsO_4_^3−^) salts. We adopted the As(V)-tolerance phenotype as a proxy to explore if the insertion of the synthetic Δ*tmk* ^GI^::*tmk_Ec_* construct brings about any noticeable physiological response to a KH_2_AsO_4_ challenge. When subjected to increasing concentrations of KH_2_AsO_4_, up to 300 mM, the growth profiles of the parental *P. putida* strain were contrastingly different from those of its Δ*tmk* ^GI^::*tmk_Ec_* derivative, *P. putida* NTW1804 (Fig. S1 in the Supplemental Material). *P. putida* NTW1804 exhibited significantly reduced growth rates and prolonged lag times at concentrations equal to and exceeding 25 mM KH_2_AsO_4_, while the two strains displayed identical growth at As(V) concentrations up to 5 mM. These observations validated the importance of the *ars1* gene cluster in As(V) tolerance, and demonstrated that the entire GI can be functionally replaced with a gene encoding the TMPK enzyme from *E. coli* without affecting the overall growth phenotype of *P. putida* KT2440. Furthermore, when *P. putida* NTW1804 was cultivated in de Bont minimal (DBM) medium under glycolytic (30 mM glucose) or gluconeogenic (30 mM citrate) conditions, or in complex LB medium, the growth phenotype was identical to that of the parental KT2440 strain (results not shown). Building on these preliminary results, we implemented the same functional replacement approach in a genome-reduced variant of *P. putida* KT2440, strain SEM10 (37). The resulting strain, *P. putida* NTW1282 (Table 1) exhibited the same growth phenotype as strain SEM10 and the engineered *P. putida* NTW1804 variant across a range of culture conditions (i.e., complex LB medium and DBM medium added with acetate, glucose or citrate; results not shown), further validating the overall strategy.

Adopting the same framework, we studied the next obvious candidate that could provide the TMPK activity. As indicated above, the very last CDS in the genes clusters encoded in the GI of strain KT2440, *PP_1964*, is predicted to encode a deoxynucleotide monophosphate kinase (i.e., dGMP/dTMP/dihydroxy-CMP kinase, dNMPK*_Pp_*). Interestingly, the entire GI could also be successfully removed by leaving only *PP_1964* under the original transcriptional and translational control sequences of the *tmk.A* fragment and translationally coupled to *holB* (Table 1). Furthermore, the resulting strain, *P. putida* SEM11, showed an almost identical growth phenotype to its parental strains EM42 and SEM10 under various culture conditions (LB medium, DBM medium supplemented with glucose, citrate, or acetate; results not shown).

**Fig 2:**
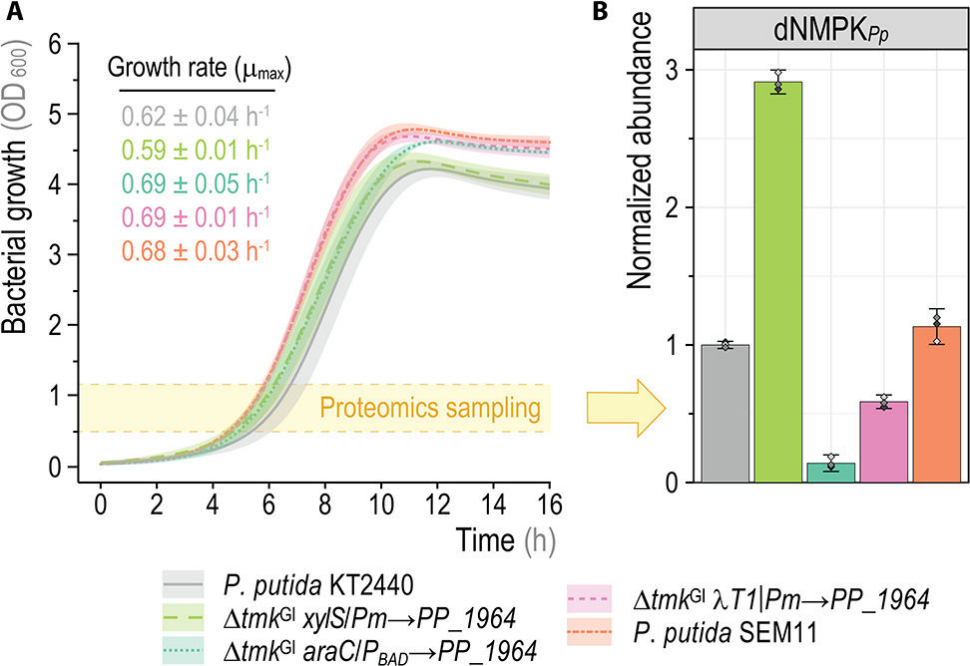
Assessing the impact of genetic modifications on thymidylate kinase function in *P. putida* KT2440. (**A**) Growth curves (smoothing splines) of the parental strain and genetic variants with a deletion in the *tmk* locus and an orthogonally regulated copy of *PP_1964*. Cells were cultured in 24-well plates in DBM medium supplemented with 30 mM glucose; cell growth was estimated as the optical density measured at 600 nm (OD_600_). Results represent average values ± standard deviations from three independent biological replicates. The yellow-shaded area indicates sampling at OD_600_ values between 0.5 and 1.2 to perform quantitative proteomics. (**B**) dNMPK*_Pp_* abundance in the parental and engineered *P. putida* strains determined via targeted quantitative proteomics. Protein abundance values were normalized to the average dNMPK*_Pp_* content obtained for strain KT2440; individual measurements are indicated for three independent biological replicates and the error bars indicate 95% confidence intervals.

In particular, using a plate reader experiment in a 24-well plate, cultures of *P. putida* KT2440 and SEM11 were grown in DBM medium with 30 mM glucose. The engineered strain displayed faster growth (10% increase in the *μ*_max_ values) and higher biomass yields (ca. 13% increase) compared to its KT2440 ancestor (Fig. 2A). Taken together, these results also provide evidence that PP_1964 (i.e., dNMPK*_Pp_*) is the *bona fide* TMPK activity of *P. putida*.

### Fine-tuning dNMPK*_Pp_* levels through synthetic transcriptional control systems reveals a high degree of buffering capacity in the nucleotide metabolism of *P. putida* KT2440

We reasoned that rendering the expression of *PP_1964* titratable through synthetic transcriptional control systems could shed light on essentiality via artificially modulating bacterial growth. Hence, the gene was placed under the control of several orthologous, inducible expression control systems. The first strategy consisted of regulating *PP_1964* expression via the XylS/*Pm* system (44), induced by 3-methylbenzoate (3-*m*Bz). Following a two-step allelic exchange approach, a synthetic construct [containing the T1 terminator from phage λ (i.e., λT1), the *xylS* gene that encodes the 3-*m*Bz-responsive regulator, the *Pm* promoter and the *PP_1964* gene] was chromosomally inserted into an intergenic region ~5.3 kb downstream of the *tmk* ^GI^ locus, followed by removal of the entire cluster but leaving *holB* intact. When cultivated in DBM medium containing 30 mM glucose, the resulting mutant strain, *P. putida* NTW1797 (Table 1), had wild-type-like growth patterns even in the absence of any chemical inducer (Fig. 2A). Indeed, the addition of 3-*m*Bz at concentrations as low as 0.1 mM resulted in a reduced biomass yield (i.e., 21% lower final OD_600_ values), and supplementing 3-*m*Bz at 1 mM negatively affected the final cell density (reduced by 26%) and *μ*_max_ (with an 11% slower growth than in the control experiment without 3-*m*Bz supplementation, Fig. S2A in the Supplemental Material). Since 3-*m*Bz is not expected to mediate toxicity in the concentration range used in these experiments (45), the detrimental effect observed could be attributed to the expression of *PP_1964*. Furthermore, considering that XylS/*Pm* is known to be a leaky expression system depending on the growth conditions (46), we followed the same allelic replacement strategy, but placed *PP_1964* under the transcriptional control of the tight L-arabinose-inducible AraC/*P_BAD_* system (47). The resulting mutant strain (*P. putida* NTW1788, Table 1) was again able to grow in DBM medium under glycolytic conditions in the absence of any inducer (Fig. 2A). The addition of L-arabinose at 0.2% (wt/vol), however, did not result in any significant increase in the *μ*_max_ and caused only minor increased in biomass yield (7%) of this engineered strain (Fig. S2B in the Supplemental Material).

Assuming that our prior attempts of making *PP_1964* conditionally dependent on the addition of inducer compounds failed due to the leakiness of the synthetic systems employed, we sought to control transcription levels via activators provided *in trans*. To this end, and concomitant with the removal of the *tmk* ^GI^ locus, we placed *PP_1964* downstream of an element composed of the T1 terminator of phage λ and the *Pm* promoter (λ*T1-Pm*). The rationale behind this design was preventing the activation of the *Pm* promoter in the absence of XylS through regulator-promoter decoupling (48). The last step of our gene editing method, resolving the previously co-integrated suicide plasmid, adopted the suicide plasmid pQURE6, which carries a copy of *xylS* and two copies of *Pm* that control the expression of the I-*Sce*I meganuclease gene as well as the replication protein gene *trfA* [i.e., making plasmid replication dependent on the addition of 3-*m*Bz (37)]. We hypothesized that if there is no transcriptional activity on *PP_1964* in the absence of XylS, the resulting mutant strain would be unable to lose pQURE6 after the procedure. However, the engineered strain (*P. putida* NTW1835, Table 1) readily lost plasmid pQURE6 regardless of 3-*m*Bz addition and showed no growth defects (Fig. 2A).

To further understand the observed phenotypes, a quantitative, whole-proteome analysis was performed on the previously described strains in the absence of any chemical inducer (Fig. 2B). The leakiness of the chromosomally-encoded XylS/*Pm* system resulted in a threefold increase of the intracellular dNMPK*_Pp_* abundance in *P. putida* NTW1797 as compared to that in strain KT2440. The AraC/*P_BAD_* system was indeed the tightest among the tested expression constructs, with an intracellular dNMPK*_Pp_* concentration of only ~20% compared to that in the parental *P. putida* strain. Surprisingly, even in the absence of the XylS activator (strain NTW1835), *PP_1964* expression from *Pm* reached ~80% of the value in the control (Fig. 2B), which may be due to transcriptional read-through from upstream CDSs. Strain SEM11 had dNMPK*_Pp_* levels comparable to that of *P. putida* KT2440 (Fig. 2B). By integrating the information about dNMPK*_Pp_* abundance and strain phenotypes gathered thus far, the following picture emerged: (i) deletion of the *tmk* ^GI^ locus and the 60 CDSs encoded therein increases biomass yields, (ii) the metabolism of *P. putida* KT2440 can tolerate a significant reduction in dNMPK*_Pp_* availability without compromising growth and (iii) a drastic increase in dNMPK*_Pp_* formation leads to reduced *μ*_max_ values and biomass yields in glucose cultures.

In a final attempt to make the formation of dNMPK*_Pp_* controllable, *PP_1964* was placed under the control of the *Pm* promoter in a different configuration without introducing *xylS* into the chromosome. In addition, because the λ *T1* terminator was suggested to have limited functionality in *P. putida* (39), the effective transcriptional termination sequence *T^S^* was placed upstream of *Pm*→*PP_1964* to prevent eventual RNA polymerase read-through. Despite these efforts, strain NTW1834 also readily lost plasmid pQURE6 (carrying *xylS*) in the absence of 3-*m*Bz and, while its growth rate was impaired (25% lower *μ*_max_), grew to the same maximum cell densities as the parental strain (Fig. S2C in the Supplemental Material). In fact, if the cells were forced to retain pQURE6 through the addition of gentamicin as well as 3-*m*Bz, the growth rate was further reduced (results not shown). In the absence of the XylS activator and the exclusion of transcriptional read-through, residual expression of *PP_1964* can be attributed to unspecific activation of *Pm* by BenR (PP_3159), which is encoded in strain KT2440 as part of the benzoate degradation pathway (49 – 51).

Thus far, the implementation of various genetic architectures around the *tmk* ^GI^ locus (Fig. S2D in the Supplemental Material) demonstrated that this large viral DNA segment contains a single essential function, providing TMPK activity with high metabolic buffering capacity. A CRISPR-Cas9-based gene disruption approach through base-editing at the single-nucleotide resolution provided definite evidence for the essentiality of dNMPK*_Pp_* in *P. putida* KT2440, as disclosed in the next section.

### CRISPR-Cas9-base-editing and complementation assays expose the essentiality of dNMPK*_Pp_* and its role as the sole thymidylate kinase of *P. putida* KT2440

**Fig 3:**
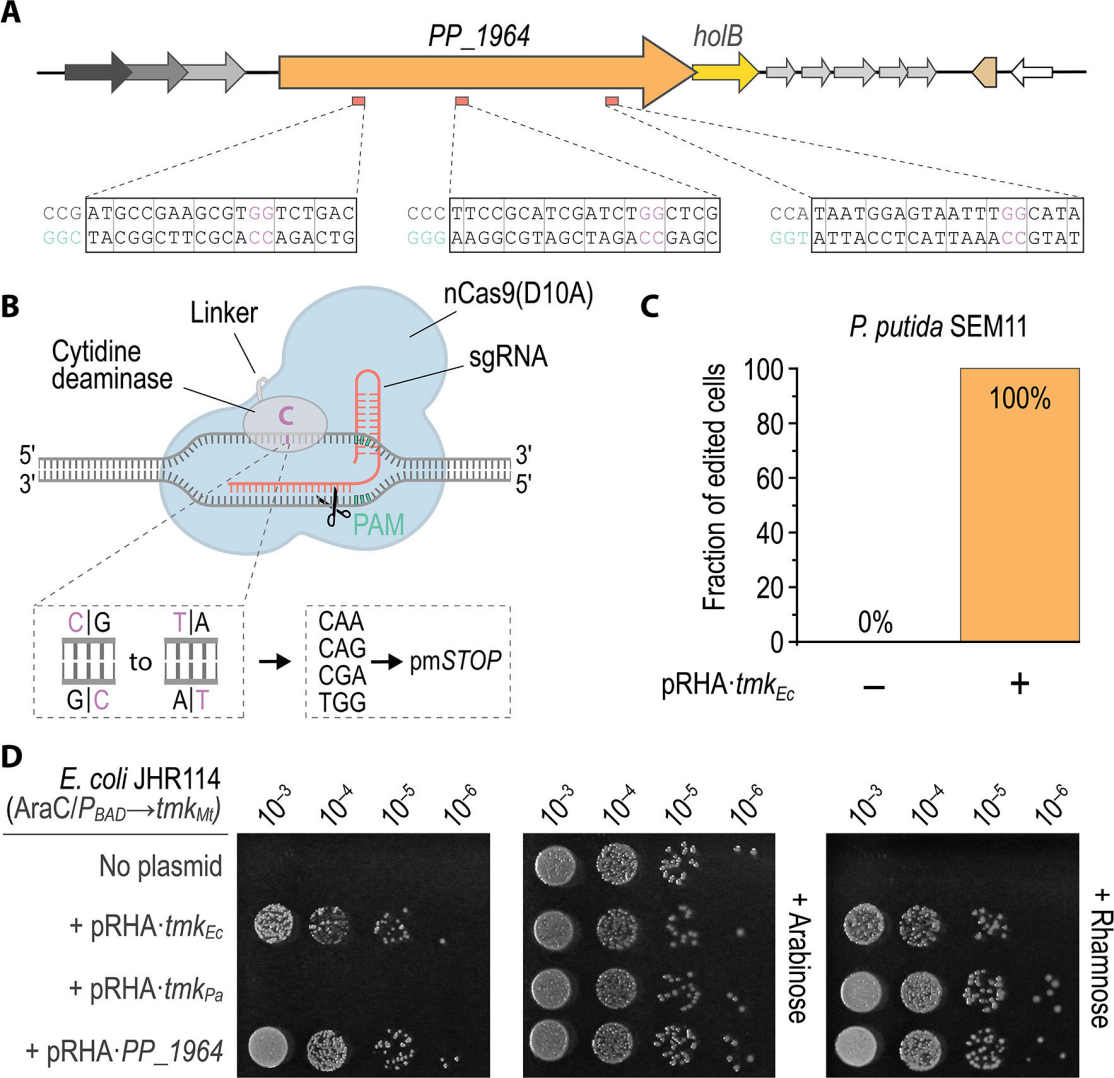
Exploring the essentiality of *PP_1964* in *P. putida* KT2440 and assessing thymidylate kinase activity of dNMPK*_Pp_*. Plasmid pMBEC6∙*PP_1964*, encoding a CRISPR-Cas9-guided cytidine base-editing system as well as three synthetic guide RNAs targeting *PP_1964*, was delivered into strain SEM11 to inactivate the dNMPK*_Pp_* gene. (**A**) Physical map indicating the locations of the three targeted protospacers within the *PP_1964* coding sequence. Target cytosine nucleotides that yield premature *STOP* codons (pm*STOP*) upon editing and transition to thymine are highlighted in magenta. Protospacer adjacent motif (PAM) sequences, 5′-*N*GG-3′ (where *N* indicates any nucleotide), are drawn in turquoise. Codon triplets are separated by vertical gray lines. (**B**) Functional principle of the CRISPR-Cas-guided cytidine base-editing system. A nicking Cas9 variant (nCas9, carrying the D10A mutation) fused to a cytidine deaminase is guided to a target locus via a synthetic guide RNA. The cytidine deaminase causes C→T transitions within a defined range in the non-target DNA strand, allowing for the introduction of pm*STOP* codons within the open reading frame (43). (**C**) Fraction of *P. putida* SEM11 clones harboring pm*STOP* codons in *PP_1964* in the presence (+) or absence (–) of the *E. coli tmk* gene (*tmk_Ec_*) after the base-editing procedure. Bar plot show the percentage of clones harboring at least one pm*STOP* codon out of 96 sequenced *P. putida* SEM11 colonies with plasmid pRHA·*tmk_Ec_* (Table 2) or the corresponding empty vector. (**D**) Droplet assay with *E. coli* strain JHR114 (Δ*tmk*) harboring a chromosomally encoded *tmk* gene from *Mycobacterium tuberculosis* under transcriptional control of the AraC/*P_BAD_* expression system (Table 2). This Tmk-deficient *E. coli* strain was transformed with derivatives of plasmid pRHA encoding either *tmk_Ec_*, *Pseudomonas aeruginosa tmk* (*tmk_Pa_*) or *PP_1964* controlled by the L-rhamnose-inducible RhaRS/*P_rhaBAD_* system. Overnight cultures, grown in tryptone medium supplemented with 0.2% (wt/vol) L-arabinose, were serially diluted with 0.9% (wt/vol) NaCl and 8 µL droplets were spotted onto solid tryptone medium supplemented with 25 µg mL^–1^ streptomycin as well as 0.2% (wt/vol) L-arabinose and 0.2% (wt/vol) L-rhamnose as indicated. Bacterial colonies were photographed after incubating the plates at 37°C for 16 h.

As illustrated by the genetic designs attempted thus far, the entire GI of *P. putida* KT2440 (including the two *tmk* gene moieties) could be successfully removed as long as either *PP_1964* was preserved or its function was complemented by a copy of the *tmk* gene from *E. coli*. Any attempts to delete *PP_1964* via allelic exchange were unsuccessful (details not shown). However, the failure of implementing these genetic manipulations is not a definite proof of gene essentiality (52). Thus, a more reliable strategy was employed to provide conclusive evidence of the role of PP_1964 as the sole source of TMPK activity in strain KT2440. To this end, *tmk_Ec_* was cloned into the expression vector pSEVA4418, in which *tmk_Ec_* was placed under the transcriptional control of the L-rhamnose-inducible RhaRS/*P_rhaBAD_* system (Table 1). In addition, plasmid pMBEC6∙*PP_1964* was constructed to introduce premature *STOP* (pmSTOP) codons via multiplexed cytidine base-editing (40). This base-editing plasmid encodes three synthetic guide RNAs that target protospacers distributed across the *PP_1964* coding sequence (Table 1) and are suitable to introduce pmSTOP codons via C-to-T transitions (Fig. 3A and B). Next, the base-editing plasmid pMBEC6∙*PP_1964* was delivered into *P. putida* SEM11 (Δ*tmk* ^GI^::*PP_1964*, Table 1) harboring either pSEVA4418 (empty vector, used as a control) or pSEVA4418∙*tmk_Ec_* (carrying the TMPK-encoding gene from *E. coli*). After transformation with pMBEC6∙*PP_1964*, recovery of the cells in liquid LB medium for 24 h and isolation of individual colonies on LB medium plates without any additives, the *PP_1964* locus was PCR-amplified and sequenced for 96 randomly picked colonies of each strain (Fig. 3C). All colonies that had received *tmk_Ec_ in trans* exhibited at least one pmSTOP codon in *PP_1964*, while none of the tested colonies without *tmk_Ec_* showed any null mutations in any of the targeted protospacer sequences. These findings provide strong evidence that the dNMPK*_Pp_* activity encoded in *PP_1964* provides the essential TMPK function in *P. putida* KT2440, as the CDS could only be inactivated if it was simultaneously complemented by *tmk_Ec_ in trans*.

To further demonstrate a role for dNMPK*_Pp_* in providing the TMPK function, a complementation assay was performed using *E. coli* strain JHR114 (Table 1), which harbors a chromosomally-encoded *tmk* gene from *Mycobacterium tuberculosis* (*tmk_Mt_*) under the control of the AraC/*P_BAD_* expression system, with its native *tmk* copy deleted (33). Hence, the growth of this strain is dependent on the induction of *tmk_Mt_* via the addition of L-arabinose, making it suitable to screen for thymidylate kinase activities provided *in trans*. JHR114 was transformed with derivatives of pRHA, harboring either *tmk_Ec_, tmk* from *Pseudomonas aeruginosa* (*tmk_Pa_*, sharing an 80.5% amino acid sequence identity with *tmk* from *P. putida* KT2440) or *PP_1964* under the transcriptional control of the L-rhamnose-inducible RhaRS/*P_rhaBAD_* system. The resulting *E. coli* strains (i.e., JHR114, and its derivatives carrying plasmids pRHA·*tmk_Ec_*, pRHA·*tmk_Pa_* or pRHA·*PP_1964*, Table 1) were pre-grown in liquid tryptone medium with L-arabinose, serially diluted and spotted onto tryptone agar with or without L-arabinose or L-rhamnose (Fig. 3D). As expected, all strains were able to grow in the presence of L-arabinose, and strain JHR114 without any plasmid was only able to grow in the presence of L-arabinose. Functional complementation of the TMPK activity by *tmk_Ec_*, *tmk_Pa_* and *PP_1964* could be verified in the presence of L-rhamnose. Surprisingly, the strains transformed with pRHA·*tmk_Ec_* and pRHA·*PP_1964* grew even in the absence of inducers, while JHR114 carrying pRHA·*tmk_Pa_* did not. In the genetic architecture of vector pRHA, *tmk_Ec_*, *tmk_Pa_*, and *PP_1964* display almost identical translation initiation rates [computed and predicted with the RBS Calculator tool (53)]—hence, different protein levels should not be the reason behind these phenotypic differences. Thus, Tmk*_Ec_* and dNMPK*_Pp_* appear to have higher TMPK activities than Tmk*_Pa_,* and the leaky transcriptional levels afforded by *P_rhaBAD_* sufficed to recover growth. When cultured in M9 minimal medium supplemented with 20 mM glucose, strain JHR114 carrying pRHA·*tmk_Pa_* was also able to grow, albeit with a severely reduced *μ*_max_ (Fig. S3 in the Supplemental Material). Addition of L-rhamnose, in contrast, restored growth for all *E. coli* strains harboring a plasmid. Interestingly, the induction of *tmk_Ec_* expression resulted in a significant growth impairment which was not observed by overexpressing *PP_1964* or *tmk_Pa_*. The results obtained from the complementation experiments in *E. coli* confirm the role of dNMPK*_Pp_* as a TMPK and expose a remarkable metabolic buffering capacity of this enzyme in bacterial hosts other than *P. putida*.

### Structural alignment and phylogenetic analysis of nucleoside monophosphate kinases

**Fig 4:**
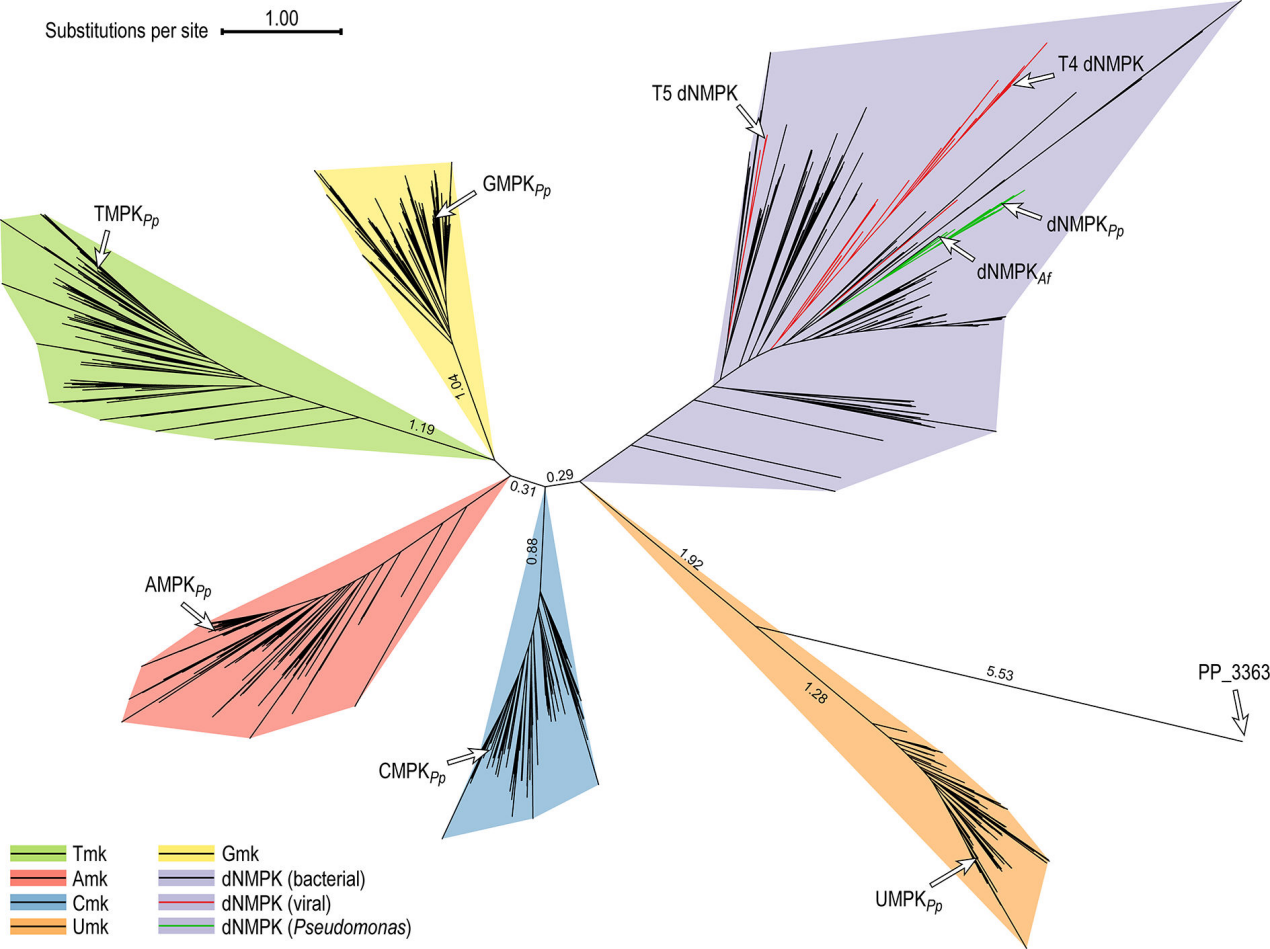
Phylogenetic tree illustrating the distribution of nucleoside monophosphate kinases in bacteria and viruses. The tree was constructed using FastTree (46) based on a structural PROMALS3D alignment (47) of 200 bacterial adenylate kinases (AMPKs), 200 bacterial guanylate kinases (GMPKs), 200 bacterial TMPKs, 200 bacterial uridylate kinases (UMPKs), and 300 enzymes annotated as (putative) deoxynucleoside monophosphate kinases in bacteria and 81 dNMPKs found in viruses. Specific cases, including enzymes of *P. putida* KT2440, are indicated with arrows; relative phylogenetic distances are indicated in the branches of the tree when relevant.

The experimental evidence presented in the preceding sections indicates that the essential *PP_1964* (dNMPK*_Pp_*) gene is the only source of TMPK activity in *P. putida* KT2440. In order to assess the evolutionary relationship of dNMPK*_Pp_* with other (deoxy)nucleoside monophosphate kinases, a phylogenetic analysis was conducted across annotated bacterial and viral genomes. To this end, we performed a structural alignment of 200 bacterial adenylate kinases (AMPKs), 200 bacterial guanylate kinases (GMPKs), 200 bacterial TMPK, 200 bacterial uridylate kinases (UMPKs), and 300 enzymes annotated as deoxynucleoside monophosphate kinase in bacteria and 81 dNMPKs found in viruses—representing a sampling space large enough to establish relationships between these functions. Next, this multi-sequence alignment was used to construct a phylogenetic tree (Fig. 4) using FastTree (54) based on the structure-based PROMALS3D alignment (55). Based on the framework proposed by Leipe et al. (56), we assumed that TMPKs belong to the heritage of a common ancestor of P-loop kinases—as this enzyme is the only (d)NMP kinase ubiquitous in Bacteria, Archaea and Eukaryota. Deoxynucleoside monophosphate kinases are closely related to the TMPK group, exemplified by the dNMPK enzyme from λ phage T4 (56 – 58). Furthermore, AMPKs and GMPKs were proposed to share a common bacterial ancestry distinct from the archaeal-eukaryotic origin of CMPK (59).

The phylogenetic tree underscores a parallel evolution of AMPK and GMPK in bacteria with a similar distance to the TMPK group—although the precise evolutionary relationships of the nucleotide kinases cannot be inferred from the analysis (60). The UMPK group, which shares no significant sequence similarity to other nucleoside monophosphate kinases (61), is clearly positioned as an outlier outside the remaining members of the tree. The enzyme encoded by *PP*_*3363*, annotated as thymidylate kinase, is even more isolated—with no apparent relationship to either the TMPK or any other group. Interestingly, the phylogenetic analysis presents a different perspective on the relationship of dNMPKs to the remaining groups, placing them in a distinct evolutionary domain. dNMPKs from both bacteria and viruses are situated closer to bacterial CMKs, which share conserved structural features with other members of the NMP kinase family (62, 63). The dNMPK sub-tree encompassing dNMPK*_Pp_* also harbors enzymes from the two archetypal *E. coli* bacteriophages T4 [i.e., Tequatrovirus T4, which recognizes dGMP, dTMP and 5-hydroxymethyl-dCMP (64)] and T5 [i.e., Tequintavirus T5, which acts on all four canonical dNMPs (65)].

Based on these observations, and to further explore the prevalence, function and evolutionary origin of enzymes in the dNMPK group of the bacterial domain, we conducted a BLASTp search (66) against non-redundant protein sequences (nrPS) using dNMPK*_Pp_* as the query and setting a maximum *E* value of 0.00001. This search identified 871 proteins with the following annotations: adenylate kinase (14 cases), deoxynucleoside monophosphate kinase (667 cases), hypothetical protein (186 cases), nucleoside triphosphate hydrolase, phosphomevalonate kinase, nucleoside triphosphate hydrolase and choline dehydrogenase or related flavoprotein (1 nrPS of each of these categories). The majority of these hits (622 nrPS) were found within the Pseudomonadales order, with additional members of the γ-proteobacteria class (totaling 696 nrPS) encountered in isolates from the Moraxellales, Enterobacterales, Oceanospirillales and Alteromonadales orders. Furthermore, orthologs of dNMPK were identified in β-proteobacteria (93 nrPS), primarily within the large Burkholderiales order (83 nrPS). Additionally, 44 homologs were identified in bacterial phages—predominantly those infecting bacteria within the Enterobacteriaceae family.

To gain an even broader understanding of the distribution and function of dNMPK enzymes, we also examined the sequenced genomes of a diverse sample of organism groups. Our analysis led to several key findings. In γ-proteobacteria, the *tmk* gene is typically found adjacent to *holB*—a genetic architecture epitomized in the *P. putida* KT2440 chromosome (Fig. 1D). We also observed that the disruption of the *tmk* gene by a GI containing a dNMPK homologue occurred in several members of γ-proteobacteria, including *Pseudomonas*, *Halomonas*, *Acinetobacter* and *Psychrobacter*—with the size of these GIs varying considerably across isolates. However, despite substantial differences in the cluster length and functional content, a strong conservation of four prophage-associated genes flanking dNMPK was found (Fig. S4 in the Supplemental Material). Moreover, we found that some strains of *E. coli*, *Shigella flexneri* or *Citrobacter freundii* contained prophages carrying potential homologs of dNMPK*_Pp_* as well as functional gene sequences for TMPK activities. These prophages were fully integrated into the genomes of their respective bacterial hosts and displayed various levels of decay. We further discovered both an intact *tmk* gene (adjacent to *holB*) and an unannotated DNA sequence in a strain of *Pseudomonas parafulva* (NS96), which, upon translation, showed 44% identity to dNMPK*_Pp_*. Interestingly, no evidence for the presence of a prophage could be found in the surrounding genomic context in this case in particular. Finally, we identified dNMPK*_Pp_* homologs located adjacent to the *holB* gene, with sequence identities between 40% and 49% to dNMPK*_Pp_* in some β-proteobacteria members, e.g., *Parapusillimonas granuli* strain DSM 18079 and *Alcaligenes faecalis* strain JQ135, and also in the γ-proteobacterium *Methylomicrobium lacus* LW14 (Fig. S4). However, no gene encoding dTMP kinase was detected in these organisms. Additionally, in these cases, no genetic elements associated with mobile genetic elements could be found in the vicinity of the dNMPK gene. The genomic position of the CDS, together with the absence of a *tmk* gene, suggests that the dNMPK enzymes have adapted to perform the essential TMPK function in these strains—possibly as a result of a unique evolutionary path (67) or a gene-loss event (68). This evolutionary adaptation bears important implications for understanding the functional versatility of dNMPK enzymes and the extent to which they can substitute for other (d)NMP kinases in different microorganisms. Interestingly, our phylogenetic tree reconstruction highlights a close relationship between dNMPK*_Pp_* and dNMPK from *A. faecalis* (dNMPK*_Af_*, Fig. 4), implying that both enzymes could display similar substrate specificities.

Drawing from the close structural relationship between dNMPK from bacteriophage T4 of *E. coli* (acting, as indicated above, on dNMP, dTMP and 5-hydroxymethyl-dCMP) and the enzyme of *P. putida* KT2440, the next section focuses on the structural aspects of dNMPK*_Pp_*, with emphasis on homology modeling and substrate-binding pocket analysis. On this basis, this part of the study aims at elucidating the potential function of the enzyme of strain KT2440 as a TMPK activity and to better understand its unique biochemical characteristics.

### Homology modeling and substrate binding pocket analysis provides structural insights of dNMPK*_Pp_*

To further explore the role of the dNMPK*_Pp_* protein as a TMPK enzyme from a structure perspective, homology modeling was performed using I-TASSER (69) with default settings and the amino acid sequence of dNMPK*_Pp_* as the entry query. The most closely related structural homologue identified was 1DEK (70), the crystal structure of bacteriophage T4 dNMPK bound to its substrates dGMP and ATP. The homology model obtained for dNMPK*_Pp_* had a template modeling score of 0.844, a root-mean-square deviation of atomic positions of 1.86 Å, an identity of 0.195 and a coverage of 0.903—indicating a reliable structural prediction (71). In addition, structure predictions of dNMPK*_Pp_* and 1DEK were made using AlphaFold (72), and the alignment of the resulting predicted structures (termed AF-dNMPK*_Pp_* and AF-1DEK, respectively) revealed a significant degree of conservation of structural features (Fig. S5 in the Supplemental Material). One key observation derived from this analysis is that both 1DEK and AF-dNMPK*_Pp_* consist of two domains of equal size. Domain 1 in 1DEK contains a five-stranded parallel β-sheet encircled by three α-helices, resembling the mononucleotide binding motif typical of P-loop-containing proteins (73). Notably, the amino acid residues forming and adjacent to the P-loop, a common component of many ATP-binding/processing proteins (74), are largely conserved between dNMPK*_Pp_* and 1DEK. Domain 2 is composed of five α-helices arranged in a myoglobin-like fold. Similarly, AF-dNMPK*_Pp_* features two distinct domains, with Domain 1 analogous to Domain 1 of 1DEK and Domain 2 adopting a myoglobin-like fold comprising five large and four smaller α-helices. In this configuration, the two domains are linked by a pair of loops, with one of these loops obstructing the substrate binding pocket identified in the 1DEK structure. The strong structural similarities between AF-dNMPK*_Pp_* and AF-1DEK suggest that the closed conformation of AF-dNMPK*_Pp_* might open upon substrate binding to form the active center of the enzyme. In fact, the positioning of the loop could indicate a narrower substrate tunnel in dNMPK*_Pp_* (Fig. S5 in the Supplemental Material), preventing larger purine nucleotides from entering the catalytic center.

**Fig 5:**
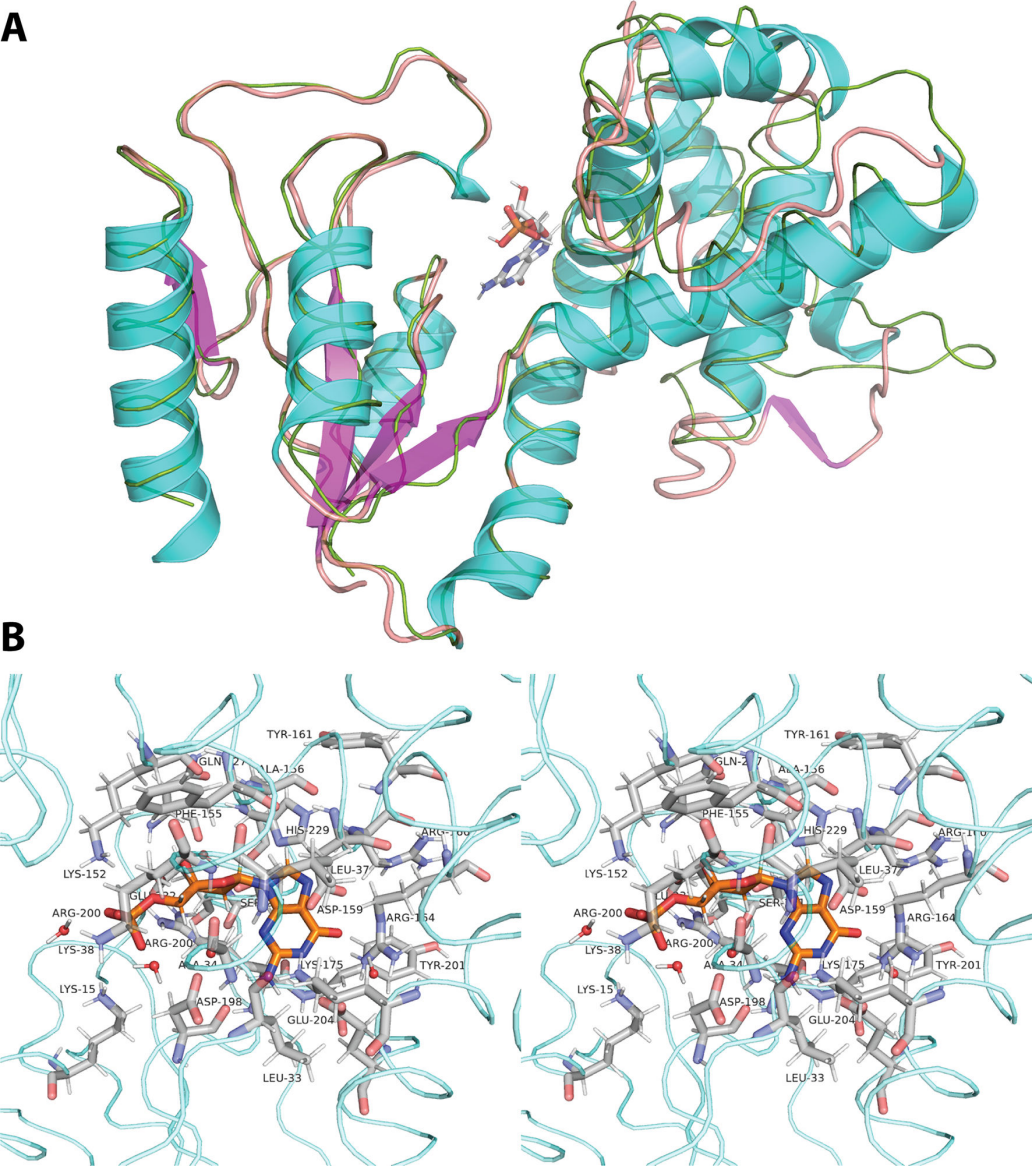
Structure of the dNMPK*_Pp_* protein of *P. putida* KT2440. The structure shown for dNMPK of *P. putida* KT2440 represents a homology model with dNMPK of *E. coli* bacteriophage T4 (1DEK). (**A**) Structure alignment of both dNMPK variants (i.e., bacterial and viral). The structure of 1DEK is indicated as a ribbon colored in green. The dNMPK*_Pp_* homology model is shown in a red cartoon, with the secondary structure elements (i.e., α-helixes and β-sheets) colored in cyan and magenta, respectively. The substrate dGMP, visible in the crystal structure of 1DEK, is shown as stick-model with atoms colored in red (oxygen), orange (phosphorus), blue (nitrogen) and gray (carbon and hydrogen). (**B**) Stereo-view of the NMP binding pocket in dNMPK*_Pp_* in complex with dGMP (front side). For a stereo-view of the back side, please refer to Fig. S6 in the Supplemental Material.

Next, we aligned the I-TASSER-predicted homology model with the 1DEK template to further examine the substrate binding pockets of dNMPK*_Pp_* (Fig. 5A). While the domain structures and the amino acid residues forming the NTP binding sites of most nucleotide-binding enzymes are largely conserved, the (d)NMP binding sites do not require a particular chain fold and can differ significantly even for the same nucleotide substrate (73). The dNMP binding pockets of 1DEK and dNMPK*_Pp_*, however, exhibit a high degree of functional consensus and sequence identity in the predicted secondary structure alignment positions (Table 2). Interestingly, some amino acids within the dNMP binding pocket in 1DEK are replaced by bulkier residues—possibly indicating a narrower substrate spectrum for dNMPK*_Pp_* (Fig. 5B; Fig. S6 in the Supplemental Material). A case in point is Val144 in 1DEK, which is replaced by Arg164 in dNMPK*_Pp_*, a modification that reduces the binding pocket’s size and potentially enables new polar interactions with thymine. Trp152 in 1DEK, part of the hydrophobic pocket, is substituted with Lys175, likely enhancing substrate specificity by excluding bulkier purines. Gln178 in 1DEK is replaced by Tyr201 in dNMPK*_Pp_*, which could afford stacking interactions with thymidine. In contrast to the observations above, the ATP binding site appears to be largely conserved between the dNMPK enzyme of bacteriophage T4 and dNMPK*_Pp_* (Fig. S7 in the Supplemental Material). Eight out of the 10 residues known to interact with the ATP substrate in the 1DEK equivalent 1DEL, which displays ATP binding (70), are identical in dNMPK*_Pp_*. The remaining three residues show only insignificant differences. Gly225 in 1DEK, substituted by Asp248 in dNMPK*_Pp_*, interacts with ATP only via its backbone carbonyl moiety; Leu227, fostering hydrophobic interactions, is replaced by its aliphatic counterpart, Ile250.

**TABLE 2:** Critical residues involved in forming the substrate binding pockets and their corresponding amino acids in *P. putida* KT2440 dNMPK*_Pp_* ^*a*^

Category and domain	1DEK residue	dNMPK*_Pp_* residue	Consensus
dNMP phosphate binding	Lys37	Lys38	**K**
	Arg68	Pro69	.
	Arg132	Lys152	+
	Arg177	Arg200	**R**
dNMP sugar binding	Met135	Phe155	h
dNMP binding pocket hydrophobic surface	Leu32	Leu33	**L**
	Ala33	Ala34	**A**
	Ile36	Leu37	l
	Trp152	Lys175	b
	Thr208	Ser231	o
	Arg177	Arg200	**R**
dNMP binding pocket locking	Asp204	Gln227	p
	His206	His229	**H**
	Thr208	Ser231	o
	Glu209	Glu232	**E**
	Gln136	Ala156	.
	Asp141	Tyr161	.
dNMP substrate specificity residues (base interactions)	Glu181	Glu204	**E**
	Arg177	Arg200	**R**
	Asp175	Asp198	**D**
	Thr140	Arg160	p
ATP/phosphate binding	Arg11	Arg12	**R**
	Gly13	Gly14	**G**
	Lys14	Lys15	**K**
	Asp15	Asp16	**D**
	Thr16	Thr17	**T**
ATP base binding	Arg197	Arg220	**R**
	Asn223	Asn246	**N**
	Gly225	Asp248	s
(hydrophobic)	Leu227	Ile250	l
(hydrophobic)	Leu230	Leu253	**L**
ATP pocket stabilization	Ser226	Thr249	o
	Ser12	Ser13	**S**

^*a*^ Conserved amino acid residues: bold and uppercase letters; *aliphatic residues* (I, V, L): l; hydrophobic residues (W, F, Y, M, L, I, V, A, C, T, H): h; *alcohol residues* (S, T): o; *polar residues* (D, E, H, K, N, Q, R, S, T): *P*; *tiny residues* (A, G, C, S): t; *small residues* (A, G, C, S, V, N, D, T, *P*): s; *bulky residues* (E, F, I, K, L, M, Q, R, W, Y): b; *positively charged residues* (K, R, H): +; *negatively charged residues* (D, E): –. A dot (·) indicates that there was no functional consensus identified at the respective position.

In summary, our homology modeling and structure predictions, together with the *in vivo* evaluated of a role for dNMPK*_Pp_* as a TMPK enzyme, highlight significant structural similarities between dNMPK*_Pp_* and the viral deoxynucleoside monophosphate kinases 1DEK/1DEL. The substitutions observed in the dNMP binding pocket may contribute to a more restricted substrate spectrum for dNMPK*_Pp_*, while the conserved ATP binding site supports similar nucleotide-attachment and phosphate transfer mechanisms between the two proteins—underlying a potential viral origin for dNMPK*_Pp_*.

## DISCUSSION

GIs, which frequently harbor functional gene clusters that convey beneficial traits to their host (75, 76), have become a target for genome streamlining strategies that aim at generating minimal-genome cell factories (77). In this sense, several studies reported genome-reduced variants of *P. putida* KT2440 with enhanced physiological properties (36, 78, 79). Increased plasmid stability and transformation efficiencies were observed owing to the removal of transposases, recombinases and restriction-modification systems as part of GIs and prophages (36)—resulting in enhanced performance in both synthetic biology setups (80) and metabolic engineering applications (81). In spite of its large size and potentially detrimental impact on genetic stability, the removal of the 65 kb long GI that disrupts the *tmk* gene of the type strain KT2440 had proven elusive so far—an occurrence suggesting that it may harbor essential functions. Building on these observations, we advanced our understanding of the essentiality and functional roles of genes encoded within the GI that disrupts the *tmk* gene in *P. putida* KT2440. Indeed, the enigma surrounding the TMPK function in this bacterium has persisted for a long time (20). Our findings open new avenues for unleashing the biotechnological potential of *P. putida* while serving as a prime case study to analyze and understand how horizontally-transferred genes evolve (82). We can now address the three competing hypotheses explaining the origin of the TMPK function. Our observations substantiate the view that the two *tmk* gene segments interrupted by the GI in strain KT2440 do not contribute independently to form a functional TMPK protein. Instead, the dNMPK gene *PP_1964*, encoded within the GI, plays a pivotal role in complementing the disrupted function. Conversely, our results do not support the hypothesis that another gene (previously identified as “thymidylate kinase,” *PP_3363*) fulfills any important role—the actual function of which is likely misannotated.

Intriguingly, the GI harboring *PP_1964* is absent in the closest phylogenetic relatives of strain KT2440, indicating that this element has been acquired relatively recently. This observation, in turn, suggests that the host metabolism has accommodated the regulation and specific activity of dNMPK*_Pp_* without significant adaptation. Indeed, the high buffering capacity observed for dNMPK*_Pp_* in both *P. putida* and *E. coli* underlines a remarkable adaptability of this enzyme in different metabolic contexts. Moreover, dNMPK*_Pp_* homologs encoded in GIs disrupting the host *tmk* gene can be found in multiple *Pseudomonas* species, as well as in representatives of the *Acinetobacter*, *Psychrobacter* and *Halomonas* families. The GI in strain KT2440 contains several genes associated with mobile genetic elements, and its viral origin has been suggested previously (20). Further evidence supporting this notion lies in the fact that the size and functional content of these GIs are highly variable, but all genome sequences analyzed have a dNMPK function within the GIs flanked by the same four orthologs. These CDSs encode a TetR/AcrR family transcriptional regulator [which are ubiquitous in Gram-negative bacteria (83)], two tyrosine-type recombinase/integrase functions [i.e., phage integrase family (84)], and one protein of unknown activity. As indicated above, GIs are known to carry functional gene clusters that bestow beneficial traits to their host—the results of our study revealed the critical role of the dNMPK gene in establishing a GI within the conserved *tmk* gene locus as a “landing site” for novel biochemical functions. Notably, we identified several strains that harbor only a dNMPK homologue and no other gene annotated to encode a TMPK function. In these bacterial strains, dNMPK, placed *in lieu* of the *tmk* gene and flanked by *mltG* (encoding a murein lytic transglycosylase) and *holB*, appears to fulfill the essential TMPK function. In addition, no genes associated with mobile genetic elements are located around the dNMPK locus. These standalone dNMPK homologs might represent the result of purifying selection, leading to the expulsion of non-essential or even deleterious genes neighboring the horizontally acquired dNMPK copy and culminating in the effective deletion of the GI. This scenario is likewise supported by the observed phylogenetic relationship with viral dNMPK proteins. Alternatively, the absence of mobile genetic elements around the dNMPK locus could suggest that such variant represents an ancestral form of this enzyme. In this case, ancestral dNMPKs might have been co-opted by mobile genetic elements such as GIs or prophages, and subsequently transferred horizontally to other bacterial strains. In the recipient bacteria, the mobile genetic elements could have inserted the dNMPK gene into the *tmk* locus—thereby resulting in the disruption of the cognate CDS. A divergent evolutionary origin of such dNMPKs is also substantiated by phylogenetic analysis. The branch of the phylogenetic tree that contains dNMPK*_Pp_* is positioned at a considerable distance from the rest of other members, displaying a remarkable diversification within the group early in the evolutionary trajectories. The properly annotated viral dNMPKs are found in clusters dispersed across different sub-trees. This rapid diversification within the dNMPK enzyme class is consistent with an evolutionary track in mobile genetic elements and viruses, which usually have a large progeny (85). Furthermore, viruses have incorporated numerous unorthodox bases, e.g., diaminopurine, preQ0 (i.e., 7-cyano-7-deazaguanine), preQ1 (i.e., pre-queuosine 1) and 5-hydroxymethyl-dCMP (86). This biochemical occurrence necessitates the evolution of enzyme structures capable of accommodating various base types and selectively convert the substrates into the target, non-canonical products.

Restoring the *tmk* sequence present in other Pseudomonads closely related to *P. putida* KT2440 led to unsuccessful outcomes. This was a surprising result, since transcriptional regulation appears functional in these cases—the placement of *holB*, *tmk_Ec_* or *PP_1964* under these control units proved effective (Fig. 1). Importantly, *P. putida* SEM11 had equal growth performance when the strain was endowed with either dNMPK*_Pp_* or TMPK*_Ec_*—suggesting that either dNMPK has no secondary role or the potentially secondary (promiscuous) activity on other NMPs do not significantly impact nucleotide pools and related biochemical processes. Removal of the entire GI harboring (among other CDSs) genes encoding two recombinases, one transposase and one integrase did not alter the growth physiology of the reduced-genome *P. putida* SEM11 compared to its predecessor SEM10 or EM42 strains—but is expected to lead to increased genome stability (79). Improved stability is a desirable trait for biotechnological applications (36), as it results in more predictable performance and limited occurrence of undesirable mutations. Furthermore, the removal of the GI may increase fitness under stressful conditions, since strain variants derived from *P. putida* KT2440 where the GI had been removed showed enhanced biomass yields and growth rates. These enhanced phenotypic features align with the known benefits of genome reduction (81), as more cellular resources are freed up for biomass formation, instead of being used for the (often, detrimental) replication of accessory chromosomal DNA segments that are not necessary under standard laboratory conditions (87). Moreover, we provide evidence supporting a significant degree of resilience in the metabolism of *P. putida*, tolerating significant changes in dNMPK*_Pp_* abundance without compromising the overall growth parameters (Fig. 2).

From a broader perspective, the adaptation of foreign DNA sequences is an unavoidable constraint as a result of horizontal gene transfer (88)—an ubiquitous mechanism that makes up a large portion of bacterial genomes (89, 90). In some cases, foreign DNA could encode functions that are immediately adapted to their recipient hosts if the competitive advantage is significant enough. Understanding how the evolution of these sequences proceeds is a major biological question and the work presented here is a decisive step in this direction. Upon transfer into a new host, the corresponding genes will tend to be mutated in an adaptation process that combines accommodation followed by assimilation (91)—resembling the concept proposed by Jean Piaget in the context of *genetic epistemology* (92). Here, “accommodation” refers to the early stage of adaptation of foreign genes into a new bacterial host, while “assimilation” is the final stage of adaptation of such genes into that host. Therefore, accommodation entails a modification of the cells' organization imposed by the resistance built up against foreign structures and processes by the native elements of the recipient bacterium. Assimilation, on the other hand, consists of progressive modification of the foreign object (in this case, the foreign TMPK activity) by the processes and structures available to the cell—a scenario well-illustrated in the present study.

In conclusion, our findings not only resolve the long-standing evolutionary riddle of the TMPK function in this bacterium but also provide valuable insights for further exploration of its biotechnological potential. These efforts also constitute a first step in the exploration of the way foreign DNAs encoding novel functions adapt to their new host—a question of major importance for synthetic biology that has remained largely underexplored. As such, this study highlights the importance of understanding the role of GIs in shaping the genetic architecture and metabolic capabilities of bacteria, with potential implications for a wide range of applications in both fundamental and applied microbiology.

## MATERIALS AND METHODS

### Bacterial strains and culture conditions

The bacterial strains employed in this study are listed in Table 1. *E. coli* and *P. putida* cultures were routinely incubated at 37°C and 30°C, respectively. For standard cultivations, cloning procedures and genome engineering manipulations, bacteria were grown in LB medium (93), containing 10  g L^−1^ tryptone, 5  g L^−1^ yeast extract and 10  g L^−1^ NaCl, pH = 7.0 (94). All liquid cultures, carried out in 250 mL Erlenmeyer flasks filled with 50 mL of the corresponding medium, were agitated at 250 rpm in a MaxQ 8000 incubator (Thermo Fisher Scientific, Waltham, MA, USA). Solid culture media contained 15 g L^−1^ agar. To select for strains carrying resistance markers, antibiotics were added to the culture media at the following concentrations: gentamicin, 10 µg mL^−1^; ampicillin, 100 µg mL^−1^; kanamycin, 50 µg mL^−1^; and streptomycin, 50 µg mL^−1^. All growth curves shown were derived from experiments carried out in 24- or 96-well microtiter plates (as indicated in the figure captions), placed in a plate reader (ELx808, BioTek Instruments; Winooski, VT, USA). To this end, *P. putida* or *E. coli* were pre-grown overnight in the respective culture medium to be used in the main experiment, diluted 1:100 (vol/vol). The cultivations were performed in DBM medium (95) buffered with 5 g L^−1^ of 3-(*N*-morpholino)propanesulfonic acid (MOPS) at pH = 7.0 (for *P. putida*) or in M9 minimal medium (for *E. coli*), each supplemented with the carbon sources and other additives indicated in the figure captions. Tryptone medium (containing 10  g L^−1^ tryptone and 10  g L^−1^ NaCl, pH = 7.0) was used in some complementation assays. Cell growth was monitored by measuring the absorbance at 630 nm; OD_600_ values were estimated from these plate-reader measurements by using correlation factors previously determined under similar cultivation conditions (96).

### Cloning procedures and plasmid construction

All plasmids used in this work are listed in Table 1. All genetic manipulations followed protocols previously established for *Pseudomonas* species (97, 98). Uracil-excision (*USER*) cloning (99, 100) was used for the construction of all plasmids in this work, except for pMBEC∙*PP_1964*, which was assembled via Golden Gate cloning (101). Oligonucleotides used to construct the plasmids and bacterial strains are listed in Table S1 in the Supplemental Material. DNA fragments employed in assembly reactions were amplified using Phusion *U* high-fidelity DNA polymerase (Thermo Fisher Scientific) according to the manufacturer’s specifications. The identity and correctness of all plasmids and DNA constructs were confirmed by Sanger sequencing (Eurofins Genomics, Ebersberg, Germany). For genotyping experiments after cloning procedures and genome manipulations, colony PCRs were performed using the commercial One*Taq* master mix (New England BioLabs, Ipswich, MA, USA), according to the manufacturer’s instructions. *E. coli* DH5α λ*pir* (Table 2) was employed for cloning procedures. Chemically competent *E. coli* cells were prepared and transformed with plasmids according to standard protocols (94); *P. putida* was rendered electro-competent following the method by Choi et al. (102). The different strategies followed for allelic replacement in the GI locus are summarized in Fig. 1D. In the “stitching” approach, the *tmk* gene of *P. putida* KT2440 shared an identical sequence with 33 CDSs encoding TMPK activities in γ-proteobacteria (accessible at the NCBI website with accession number WP_020192642.1).

### Mass spectrometry-aided targeted proteomics

Overnight pre-cultures of *P. putida* strains were grown in DBM medium supplemented with 30 mM glucose. The main cultures, consisting of 25 mL of the same medium, were prepared in 250 mL Erlenmeyer flasks and inoculated with an initial OD_600_ of 0.05. During the mid-exponential phase (Fig. 2A), samples were collected and cells were harvested through centrifugation at 17,000 × *g* for 2 min at 4°C. Following supernatant removal, the cell pellets were frozen and stored at –80°C until quantitative proteomic analysis, conducted as previously described (103, 104). A protein database comprised of the *P. putida* reference proteome [UP000000556 (20)] was utilized to assign the detected peptides to their respective functions, along with heterologously expressed proteins. Proteomic data obtained as described above were analyzed using a customized R script within RStudio (version 2021.09.2). Initially, only entries with complete values were retained, filtering for proteins detected in every replicate of at least one condition. The abundance values were log_2_-transformed and normalized via variance stabilization normalization using the versatile *vsn* package (105). Subsequently, the dNMPK*_Pp_* abundance in the experimental strains tested was extracted from the data set and represented graphically after normalization against the protein content of *P. putida* strain KT2440 (104).

### Phylogenetic analysis and construction of phylogenetic trees

Amino acid sequences of enzymes belonging to the group of nucleoside monophosphate kinases were extracted from Uniprot (106). Queries were used with the respective protein EC numbers and “*Bacterium*” or “*Viruses*” as taxonomy identifiers. Entries for AMPK, CMPK, GMPK, TMPK and UMPK were filtered for “reviewed” entries. A filter was applied to retain only proteins with a length between 180 and 300 amino acids; proteins with identical sequences were removed. Then, a sample of proteins for each functional class was randomly drawn as indicated in the text and in the figure captions. After this sampling, the sequences of proteins found *in P. putida* KT2440 were added to the data sets. A single *FASTA* file, containing all sampled protein sequences, was used to perform a structural alignment via Promals3D (55) using the standard settings. The alignment in FASTA format was trimmed to remove gaps not present in at least 10% of all sequences using *trimAl* version 1.2 (107). The phylogenetic tree was constructed with *FastTree* version 2.1.12 (54) using the command (FastTree.exe -quote -wag -cat 20 alignment_trimmed.fasta).

### Data and statistical analysis

All experiments were performed with at least three independent biological replicates unless otherwise stated, and the mean values ± standard deviation are presented. Maximum exponential growth rates (*μ*_max_) and maximum absorbance increases (as an indication of biomass yield) were determined via linear regression on log-transformed growth data using the web-tool *QurvE* (108).

## SUPPLEMENTAL MATERIAL

Supplemental Information mbio.01081-23-s0001.pdf Table S1 and Fig. S1 to S7.

ASM does not own the copyrights to Supplemental Material that may be linked to, or accessed through, an article. The authors have granted ASM a non-exclusive, world-wide license to publish the Supplemental Material files. Please contact the corresponding author directly for reuse.
