# Supplementary material for: Recursive genome engineering decodes the evolutionary origin of an essential thymidylate kinase activity in Pseudomonas putida KT2440
Source: mBio. 2023 Sep 21;14(5):e01081-23. doi: 10.1128/mbio.01081-23 (PMC10653934; doi:10.1128/mbio.01081-23)
Supplement: Supplemental Information — Table S1 and Fig. S1 to S7. [file mbio.01081-23-s0001.pdf]

SUPPLEMENTAL MATERIAL

Recursive genome engineering decodes the evolutionary origin of an essential thymidylate kinase activity in *Pseudomonas putida* KT2440

Table S1. Oligonucleotides used in this study.

Name	Sequence (5'→3')	Application	Template	Product
pSNW-*USER*_F	AGTCGACCUGCAGGCATGCAAGCTTCT	Linearization of suicide vector pSNW2	Vector pSNW2 (1)	Linearized pSNW2
pSNW-*USER*_R	AGGATCUAGAGGATCCCCGGGTACCG	Linearization of suicide vector pSNW2	Vector pSNW2 (1)	
stitch_*tmk*_HA1_F	AGATCCUCGCAGCAAGAGGCCAAG	Amplification of HA1 for the deletion of *tmk*^{GI} to restore *tmk*	*P. putida* KT2440 genomic DNA	pSNW2·stich-*tmk*
stitch_*tmk*_HA1_R	AGGTGGCGUCCGTGAAACGGTCACACAGC	Amplification of HA1 for the deletion of *tmk*^{GI} to restore *tmk*	*P. putida* KT2440 genomic DNA	
stitch_*tmk*_HA2_F	ACGCCACCUATGCCTATCAG	Amplification of HA1 for the deletion of *tmk*^{GI} to restore *tmk*	*P. putida* KT2440 genomic DNA	
stitch_*tmk*_HA2_R	AGGTCGACUAAGCAACCACCGTCTGAAC	Amplification of HA1 for the deletion of *tmk*^{GI} to restore *tmk*	*P. putida* KT2440 genomic DNA	
del-*tmk*_HA1_F	AGATCCUGGTATGATGCTGCAGACCG	Construction of pSNW2·Δ*tmk*^{GI} and pSNW2·Δ*tmk*^{GI}::*tmk*$_{Ec}$	*P. putida* KT2440 genomic DNA	pSNW2·Δ*tmk*^{GI}
del-*tmk*_HA1_R	ACAGGCAGUCCTTAATGTTGTTC	Construction of pSNW2·Δ*tmk*^{GI} and pSNW2·Δ*tmk*^{GI}::*tmk*$_{Ec}$	*P. putida* KT2440 genomic DNA	
del-*tmk*_HA2_F	ACTGCCTGUGGCTGAGGCCTACCCT	Construction of pSNW2·Δ*tmk*^{GI} and pSNW2·Δ*tmk*^{GI}::*tmk*$_{Ec}$	*P. putida* KT2440 genomic DNA	
del-*tmk*_HA2_R	AGGTCGACUCTGGGCCAGACTGGG	Construction of pSNW2·Δ*tmk*^{GI} and pSNW2·Δ*tmk*^{GI}::*tmk*$_{Ec}$	*P. putida* KT2440 genomic DNA	
ins-*tmk*$_{Ec}$ _HA1_R	ATAGGCAGUCCTTAATGTTGTTCG	Construction of pSNW2·Δ*tmk*^{GI}::*tmk*$_{Ec}$	*P. putida* KT2440 genomic DNA	pSNW2·Δ*tmk*^{GI}::*tmk*$_{Ec}$
tmk$_{Ec}$_F	ACTGCCTAUGCGCAGTAAGTATATCGTCAT	Construction of pSNW2·Δ*tmk*^{GI}::*tmk*$_{Ec}$	*E. coli* MG1655 genomic DNA	
tmk$_{Ec}$ _R	AGCCATCAUGCGTCCAACTCCTTCAC	Construction of pSNW2·Δ*tmk*^{GI}::*tmk*$_{Ec}$	*E. coli* MG1655 genomic DNA	
ins-*tmk*$_{Ec}$ _HA2_F	ATGATGGCUGAGGCCTACCCTTGG	Construction of pSNW2·Δtmk^{GI}::*tmk*$_{Ec}$	*P. putida* KT2440 genomic DNA	
LP_HA1_F	AGATCCUCAAGGCCAATGACCTTCTGG	Construction of pSNW2·*xylS*/*Pm*→*PP_1964*	*P. putida* KT2440 genomic DNA	pSNW2·*xylS*/*Pm*→*PP_1964*
LP_HA1_R	ATCCCTGUGTTCAACCACCGATG	Construction of pSNW2·*xylS*/*Pm*→*PP_1964*	*P. putida* KT2440 genomic DNA	
SEVA-*cargo*_F	ACAGGGAUCTCAAGAAGATCCTTTGATCTT	Construction of pSNW2·*xylS*/*Pm*→*PP_1964*	Vector pS628(BCD2)→*msfGFP* (2)	
SEVA-*xylS-Pm*_R	ATATGTUTTTCCTCCTAAACATGCG TGCATAAAGCCTAAGGGGTAGG	Construction of pSNW2·*xylS*/*Pm*→*PP_1964*	Vector pS628(BCD2)→*msfGFP* (2)	pSNW2·*xylS*/*Pm*→*PP_1964*
*PP_1964*_for_LP_F	AACATAUGCCAACCATCATCGGCCTC	Construction of pSNW2·*xylS*/*Pm*→*PP_1964*	*P. putida* KT2440 genomic DNA	
*PP_1964*_for_LP_R	ACGTTAAUCGACGCGAACGCAGCT	Construction of pSNW2·*xylS*/*Pm*→*PP_1964*	*P. putida* KT2440 genomic DNA	
LP_HA2_F	ATTAACGUCGTAGGAGCGGGTTTAC	Construction of pSNW2·*xylS*/*Pm*→*PP_1964*	*P. putida* KT2440 genomic DNA	
LP_HA2_R	AGGTCGACUGATCCGCACCACCTACTC	Construction of pSNW2·*xylS*/*Pm*→*PP_1964*	*P. putida* KT2440 genomic DNA	

open_pSNW2·*xylS*/*Pm*→*PP_1964*_F	ACGGGTGGCUTGATTACGAACGTTTAATTAA	Linearization of pSNW2·*xylS*/*Pm*→*PP_1964* to insert *araC*/P_{araB}	Vector pSNW2·*xylS*/*Pm*→*PP_1964*	pSNW2·*araC*/P_{araB}→*PP_1964*
open_pSNW2·*xylS*/*Pm*→*PP_1964*_R	ACCCCGCAUGTTTAGGAGGAAAAACATATG	Linearization of pSNW2·*xylS*/*Pm*→*PP_1964* to insert *araC*/P_{araB}	Vector pSNW2·*xylS*/*Pm*→*PP_1964*	
araC/P_{araB}_F	AGCCACCCGUCAAGCCGTCAATTGTC	Amplification of *araC*/P_{araB}	Vector pPS39 (3)	
araC/P_{araB}_R	ATGCGGGGUACCGAGCTCGAATTC	Amplification of *araC*/P_{araB}	Vector pPS39 (3)	
del_*xylS*_F	AGGAGTUGCAAGAAGCGGATACAGGAG	Linearization of pSNW2·*xylS*/*Pm*→*PP_1964* to remove *xylS*	Vector pSNW2·*xylS*/*Pm*→*PP_1964*	pSNW2·*Pm*→*PP_1964*
del_*xylS*_R	AACTCCUGAGTTGCCTTCCGACACCC	Linearization of pSNW2·*xylS*/*Pm*→*PP_1964* to remove *xylS*	Vector pSNW2·*xylS*/*Pm*→*PP_1964*	
open-pSNW2·*Pm*→*PP_1964*_F	ATGTTTGCAUTGACGCAGGGTGTCG	Linearization of pSNW2·*Pm*→*PP_1964* to replace λ*T1* with the strong terminator T^S	Vector pSNW2·*Pm*→*PP_1964*	pSNW2·T^S|*Pm*→*PP_1964*
open-pSNW2·*Pm*→*PP_1964*_R	ATGCGCTCGGUTTACCCGCGAAGAAGCC	Linearization of pSNW2·*Pm*→*PP_1964* to replace λ*T1* with the strong terminator T^S	Vector pSNW2·*Pm*→*PP_1964*	
T^S_F	ACCGAGCGCAUGCTCGAGTACTTC	Amplification of T^S	Synthetic oligonucleotide	
T^S_R	ATGCAAACAUCCGGCTCTGTCACAGC	Amplification of T^S	Synthetic oligonucleotide	
del-*PP_1964*_HA1_F	AGATCCUCGCTTGCCAATACCCTG	Amplification of HA1 for the deletion of *PP_1964*	*P. putida* KT2440 genomic DNA	pSNW2·Δ*PP_1964*
del-*PP_1964*_HA1_R	ATTCATTTTTGGCTCCAGGCCC	Amplification of HA1 for the deletion of *PP_1964*	*P. putida* KT2440 genomic DNA	
del-*PP_1964*_HA2_F	AATGAAUCCGCTTGGAGCTGCGT	Amplification of HA2 for the deletion of *PP_1964*	*P. putida* KT2440 genomic DNA	
del-*PP_1964*_HA2_R	AGGTCGACUGGCTCTGCTTGGTCAACAG	Amplification of HA2 for the deletion of *PP_1964*	*P. putida* KT2440 genomic DNA	
del-*tmk.A*_HA1_F	AGATCCUGGTATGATGCTGCAGACCG	Amplification of HA1 for the deletion of *tmk.A*	*P. putida* KT2440 genomic DNA	pSNW2·Δ*tmk.A*
del-*tmk.A*_HA1_R	ACAGGCAGUCCTTAATGTTGTTC	Amplification of HA1 for the deletion of *tmk.A*	*P. putida* KT2440 genomic DNA	
del-*tmk.A*_HA2_F	ACTGCCTGUGAGCTAGGTTTGGTCGC	Amplification of HA2 for the deletion of *tmk.A*	*P. putida* KT2440 genomic DNA	
del-*tmk.A*_HA2_R	AGGTCGACUTCTGACATAGCTGCCTCCG	Amplification of HA2 for the deletion of *tmk.A*	*P. putida* KT2440 genomic DNA	
*PP_1964*_STOPspacer_1_F	ATCGAGGTCTCCGTGGGTCAGACCACGCTTC GGCATGTTTTAGAGCTAGAAATAGC	Amplification of a gRNA fragment to construct plasmid pMBEC6·*PP_1964*	Vector pEX128·gRNA	pMBEC6·*PP_1964*
*PP_1964*_STOPspacer_1_R	ATCGAGGTCTCCCTCGTTTCTTAGCTGCCTA TACGG	Amplification of a gRNA fragment to construct plasmid pMBEC6·*PP_1964*	Vector pEX128·gRNA	
*PP_1964*_STOPspacer_2_F	ATCGAGGTCTCCCGAGCCAGATCGATGCGG AAGTTTTAGAGCTAGAAATAGC	Amplification of a gRNA fragment to construct plasmid pMBEC6·*PP_1964*	Vector pEX128·gRNA	
*PP_1964*_STOPspacer_2_R	ATCGAGGTCTCCCATATTTCTTAGCTGCCTAT ACGG	Amplification of a gRNA fragment to construct plasmid pMBEC6·*PP_1964*	Vector pEX128·gRNA	
*PP_1964*_STOPspacer_3_F	ATCGAGGTCTCCTATGCCAAATTACTCCATTA GTTTTAGAGCTAGAAATAGC	Amplification of a gRNA fragment to construct plasmid pMBEC6·*PP_1964*	Vector pEX128·gRNA	
*PP_1964*_STOPspacer_3_R	ATCGAGGTCTCCAAACTTTCTTAGCTGCCTAT ACGG	Amplification of a gRNA fragment to construct plasmid pMBEC6·*PP_1964*	Vector pEX128·gRNA	

open-pSNW2·$\Delta tmk^{Gl}::tmk_{Ec}$_F	ATTAATGGCUGAGGCCTACCCTTG	Linearization of plasmid pSNW2·$\Delta tmk^{Gl}::tmk_{Ec}$ to replace tmk_{Ec} with *PP_1964*	Vector pSNW2·$\Delta tmk^{Gl}::tmk_{Ec}$	pSNW2·Δtmk^{Gl}::*PP_1964*
open-pSNW2·$\Delta tmk^{Gl}::tmk_{Ec}$_R	ATAGGCAGUCCTTAATGTTGTTCG	Linearization of plasmid pSNW2·$\Delta tmk^{Gl}::tmk_{Ec}$ to replace tmk_{Ec} with *PP_1964*	Vector pSNW2·$\Delta tmk^{Gl}::tmk_{Ec}$	
*PP_1964*_F	ACTGCCTAUGCCAACCATCATCGGCC	Amplification of *PP_1964*	*P. putida* KT2440 genomic DNA	
*PP_1964*_R	AGCCATTAAUCGACGCGAACGCAGC	Construction of plasmid pSNW2·Δtmk^{Gl}::*PP_1964*	*P. putida* KT2440 genomic DNA	
del-*PP_3363*_HA1_F	AGATCCUCGAATTCGAGGATTGCCGTG	Amplification of HA1 for the deletion of *tmk.A*	*P. putida* KT2440 genomic DNA	pSNW2·Δ*PP_3363*
del-*PP_3363*_HA1_R	ACATCGUGCAATACCAGTTGGC	Amplification of HA1 for the deletion of *tmk.A*	*P. putida* KT2440 genomic DNA	
del-*PP_3363*_HA2_F	ACGATGUGAGGCCTGGGTGAGTACG	Amplification of HA2 for the deletion of *tmk.A*	*P. putida* KT2440 genomic DNA	
del-*PP_3363*_HA2_R	AGGTCGACUGACGGCAAGCTTATCAAGGG	Amplification of HA2 for the deletion of *tmk.A*	*P. putida* KT2440 genomic DNA	

Sequences used for the construction of the same plasmid *via* USER assembly (4) or Golden Gate cloning (5) are shaded in the same tone; primers used for USER assembly contain U residues as indicated.

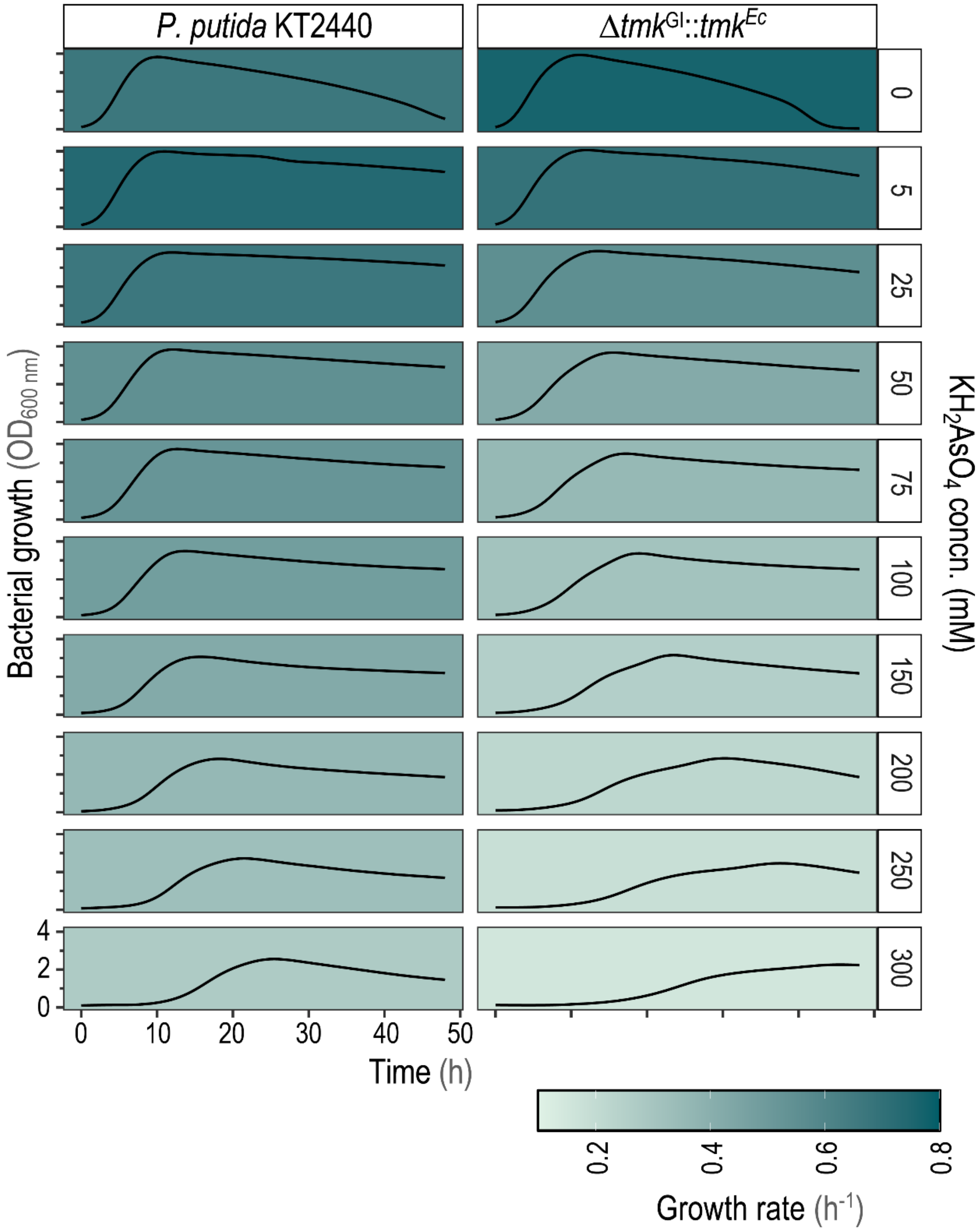

Fig. S1 · As(V) tolerance in wild-type and engineered *P. putida* strains. Cells were cultured in microtiter plate readers in de Bont minimal medium supplemented with 20 mM glucose and varying concentrations (concn.) of KH_2AsO_4 as indicated in the diagram. The experiments were carried out with **(A)** wild-type *P. putida* KT2440 and **(B)** its Δ*tmk*^{GI}::*tmk*$_{Ec}$ derivative, *P. putida* NTW1701.

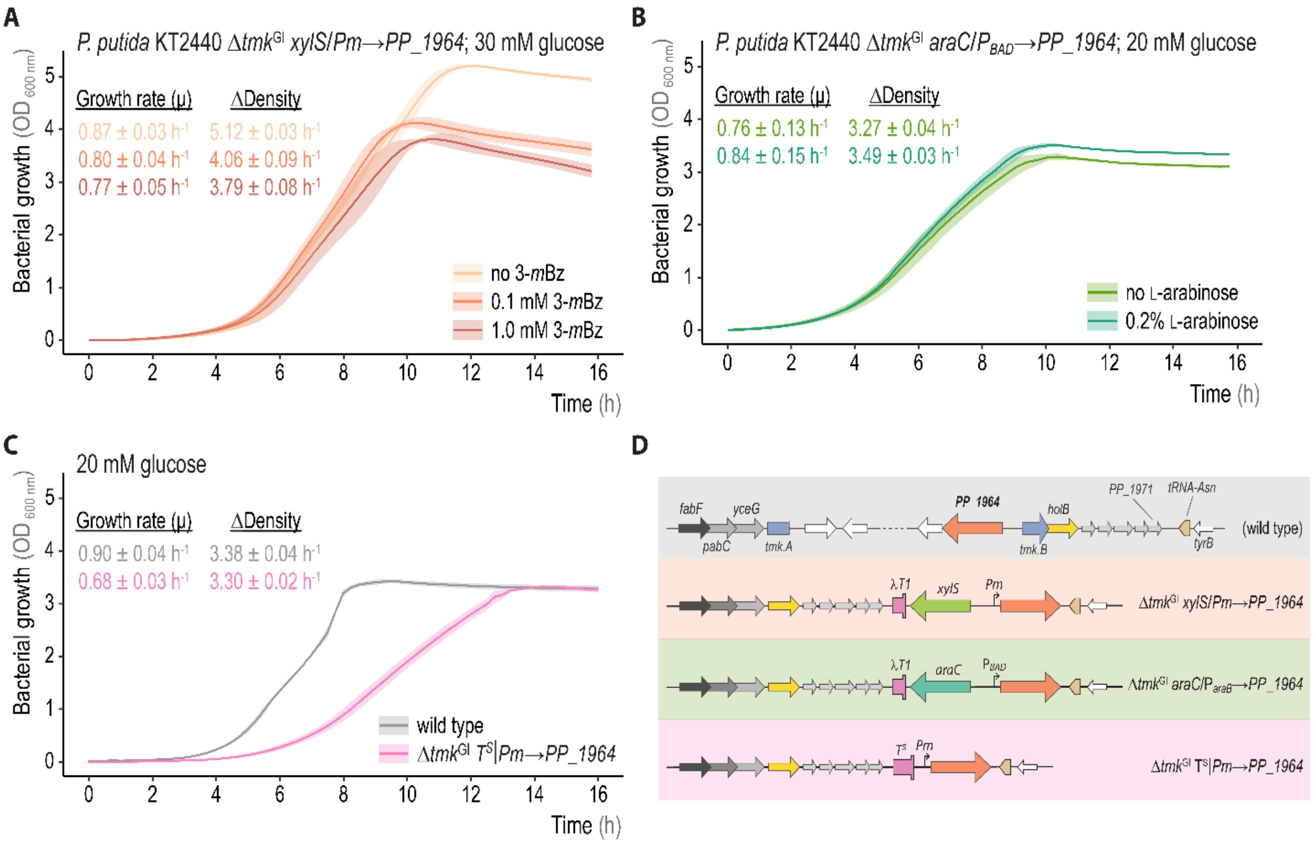

Fig. S2 · Effect of genetic context of *PP_1964* and chemical inducers on the growth of engineered *P. putida*. The data in panels **(A)** to **(C)** represent optical density measured at 600 nm ($OD_{600\ nm}$)-converted absorption measurements from 96-well microtiter plate experiments. In all of these experiments, cells were cultured in microtiter plate readers in de Bont minimal medium supplemented with 30 mM glucose and varying concentrations of chemical inducers as indicated. **(A)** Growth of a *P. putida* strain variant in which *PP_1964* was artificially regulated by the XylS/*Pm* system, induced by 3-methylbenzoate (3-*m*Bz), and the *tmk*^{CI} segment was removed from the chromosome. Mean values ± standard deviation from three biological replicates are shown in the diagram. **(B)** Growth of a *P. putida* strain variant in which *PP_1964* was controlled by the L-arabinose–inducible AraC/P_{BAD} expression system in addition to removal of the *tmk*^{CI} segment. Mean values ± standard deviation from five biological replicates are shown in the diagram. **(C)** Growth of wild-type strain KT2440 and a strain derivative in which the strong terminator sequence T^S was placed upstream of a chromosomally-encoded *Pm*→*PP_1964* element in the absence of *xylS* or any chemical inducer. Mean

values ± standard deviation from three biological replicates are shown in the diagram. **(D)** Overview of the genetic architecture around the *tmk*^{Cl} locus in all of the tested strains (please refer to **Table 1** for further details on the strains genotype).

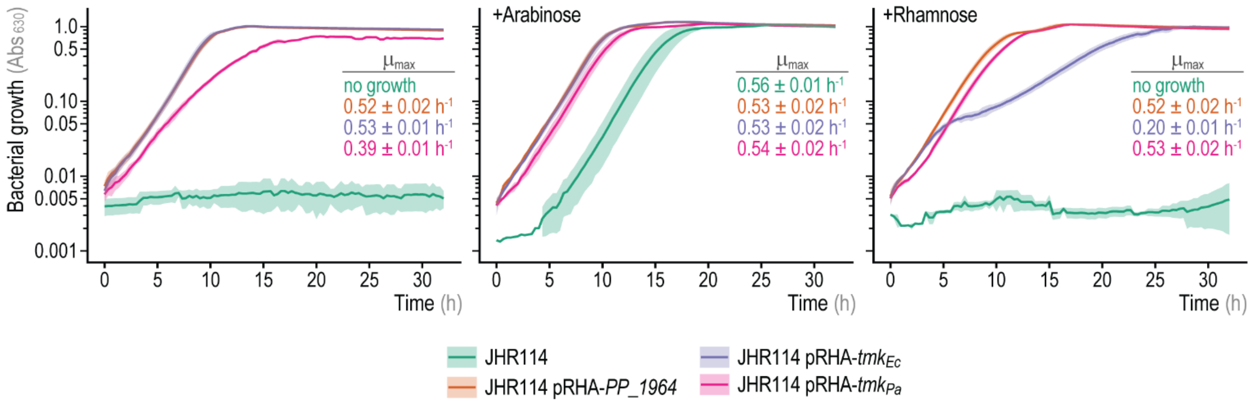

Fig. S3 · Tmk complementation assays in *E. coli* JHR114. The selection *E. coli* strain was transformed with derivatives of plasmid pRHA-CDC8 encoding either tmk_{Ec}, *Pseudomonas aeruginosa tmk* (tmk_{Pa}) or the native *PP_1964* gene controlled by the L-rhamnose–inducible RhaRS/P_{rhaBAD} expression system (please refer to **Table 1** for further details on the strains genotype). Overnight cultures, grown in LB medium supplemented with 0.2% (w/v) L-arabinose, were diluted 1:100 in M9 minimal medium supplemented with 20 mM glucose and 0.2% (w/v) L-arabinose or 0.2% (w/v) L-rhamnose as indicated. Cultures were incubated in a 96-well microtiter plate with periodic measurement of the absorbance at 630 nm (Abs_{630}). Mean values ± standard deviation from five biological replicates are shown in the diagram.

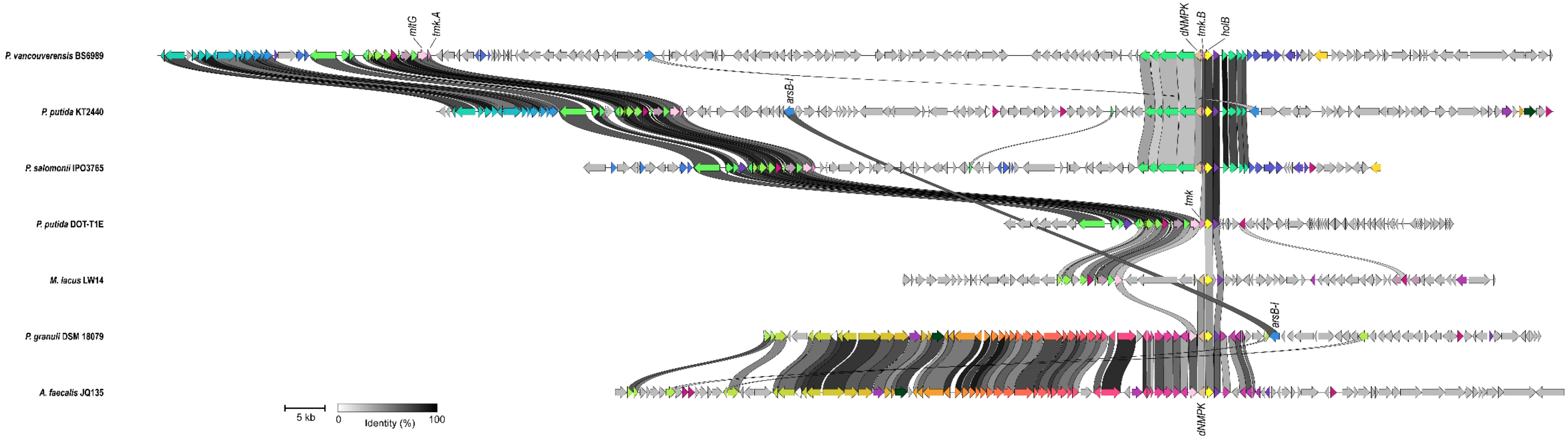

Fig. S4 · Alignment of the genetic context around the dNMPK gene in different species. The gene clusters are centered to the essential *holB* gene. The top three strains shown in the diagram (i.e. *Pseudomonas vancouverensis* BS6989, *P. putida* KT2440 and *Pseudomonas salomonii* iPO3765) have a *tmk* gene disrupted by a genetic island of varying size. *P. putida* DOT-T1E was added as a reference pseudomonad with an intact *tmk* and without any other dNMPK-encoding gene. In the bottom three strains (i.e. *Methylomicrobium lacus* LW14, *Parapusillimonas granuli* strain DSM 18079 and *Alcaligenes faecalis* strain JQ135), no *tmk* homologue could be identified and, instead, a dNMPK gene flanks *holB*. Genome sequences were retrieved from the *Pseudomonas* database (6) and other bacterial genome databases (7); the figure was created using *clinker* (8). Homologous genes are highlighted with identical fill colors.

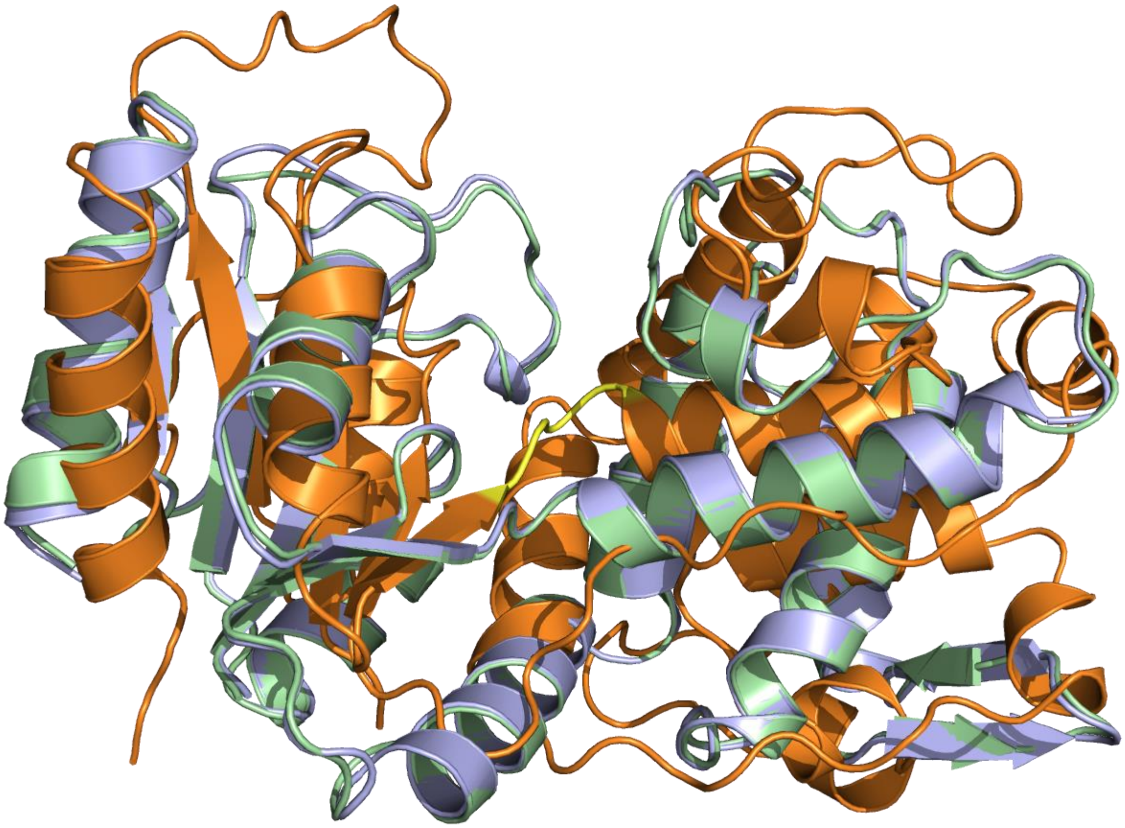

Fig. S5 · Structure alignment of the AlphaFold-predicted structure for dNMPK$_{Pp}$. The dNMPK$_{Pp}$ structure (orange) was imposed over that of the *E. coli* bacteriophage T4 dNMPK (blue), as well as the crystal structure of T4 dNMPK (1DEK, green). The loop obstructing the substrate binding pocket in the AlphaFold-predicted dNMPK$_{Pp}$ structure is highlighted in yellow. The analysis and graphical display was executed with AlphaFold (9).

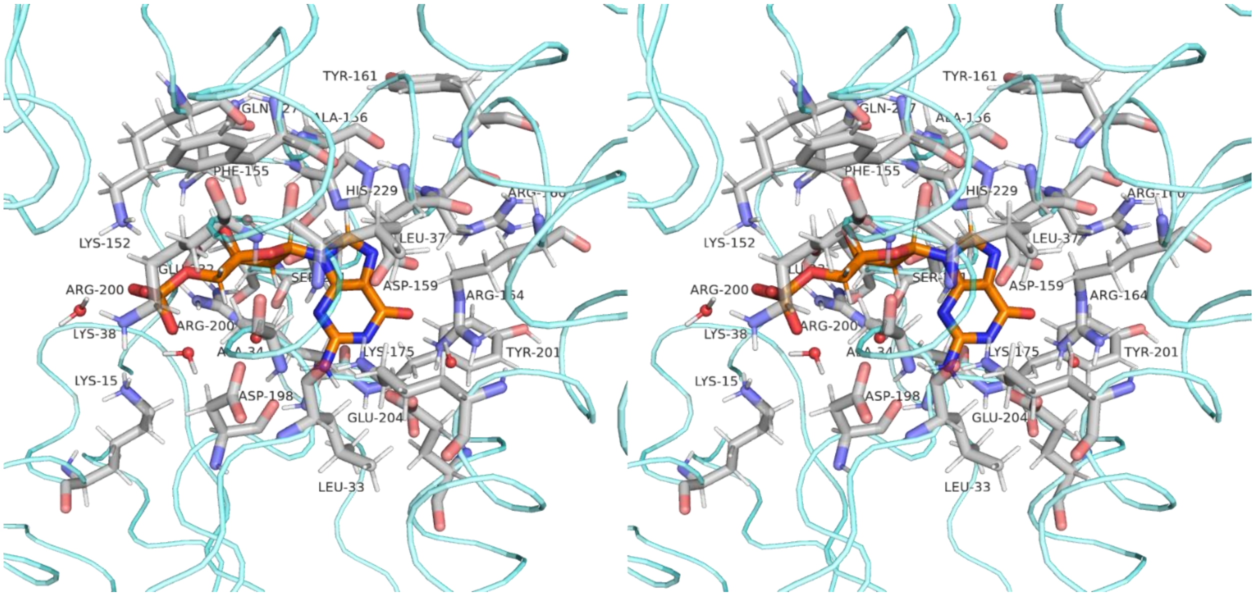

Fig. S6 · Stereo-view of the NMP binding pocket in dNMPK$_{Pp}$ in complex with dGMP (back side). The structure shown represents a homology model with dNMPK from the *E. coli* bacteriophage T4 (1DEK).

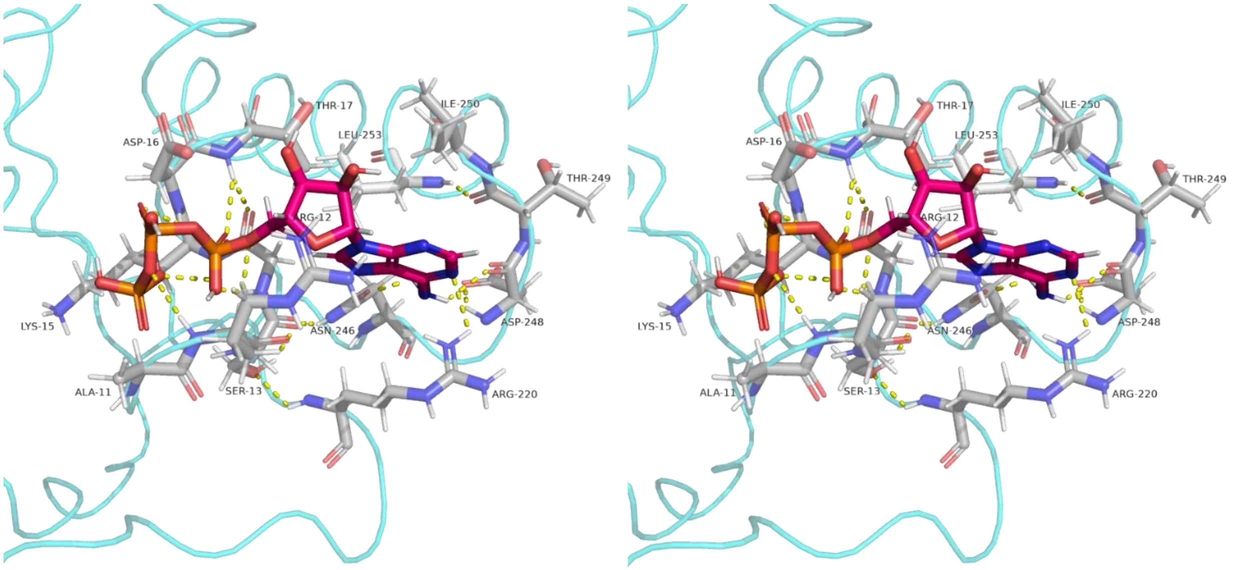

Fig. S7 · Stereo-view of the NTP binding pocket in dNMPK$_{Pp}$ in complex with ATP (back side). The structure shown represents a homology model with dNMPK from the *E. coli* bacteriophage T4 (1DEK).

Supplemental References

1. Wirth NT, Kozaeva E, Nikel PI. 2020. Accelerated genome engineering of *Pseudomonas putida* by I-*Sce*I—mediated recombination and CRISPR-Cas9 counterselection. *Microb Biotechnol* 13:233-249. https://doi.org/10.1111/1751-7915.13396.
2. Wirth NT, Nikel PI. 2021. Combinatorial pathway balancing provides biosynthetic access to 2-fluoro-*cis,cis*-muconate in engineered *Pseudomonas putida*. *Chem Catal* 1:1234-1259. https://doi.org/10.1016/j.checat.2021.09.002.
3. Calero P, Jensen SI, Nielsen AT. 2016. Broad-host-range *ProUSER* vectors enable fast characterization of inducible promoters and optimization of *p*-coumaric acid production in *Pseudomonas putida* KT2440. *ACS Synth Biol* 5:741-753. https://doi.org/10.1021/acssynbio.6b00081.
4. Nour-Eldin HH, Geu-Flores F, Halkier BA. 2010. *USER* cloning and *USER* fusion: The ideal cloning techniques for small and big laboratories. *Methods Mol Biol* 643:185-200. https://doi.org/10.1007/978-1-60761-723-5_13.
5. Pryor JM, Potapov V, Kucera RB, Bilotti K, Cantor EJ, Lohman GJS. 2020. Enabling one-pot *Golden Gate* assemblies of unprecedented complexity using data-optimized assembly design. *PLoS One* 15:e0238592. https://doi.org/10.1371/journal.pone.0238592.
6. Winsor GL, Griffiths EJ, Lo R, Dhillon BK, Shay JA, Brinkman FS. 2016. Enhanced annotations and features for comparing thousands of *Pseudomonas* genomes in the *Pseudomonas* genome database. *Nucleic Acids Res* 44:D646-653. https://doi.org/10.1093/nar/gkv1227.
7. Karp PD, Billington R, Caspi R, Fulcher CA, Latendresse M, Kothari A, Keseler IM, Krummenacker M, Midford PE, Ong Q, Ong WK, Paley SM, Subhraveti P. 2019. The BioCyc collection of microbial genomes and metabolic pathways. *Brief Bioinform* 20:1085-1093. https://doi.org/10.1093/bib/bbx085.
8. Gilchrist CLM, Chooi YH. 2021. *clinker* & *clustermap.js*: automatic generation of gene cluster comparison figures. *Bioinformatics* 37:2473-2475. https://doi.org/10.1093/bioinformatics/btab007.
9. Jumper J, Evans R, Pritzel A, Green T, Figurnov M, Ronneberger O, Tunyasuvunakool K, Bates R, Žídek A, Potapenko A, Bridgland A, Meyer C, Kohl SAA, Ballard AJ, Cowie A, Romera-Paredes B, Nikolov S, Jain R, Adler J, Back T, Petersen S, Reiman D, Clancy E, Zielinski M, Steinegger M, Pacholska M, Berghammer T, Bodenstein S, Silver D, Vinyals O, Senior AW, Kavukcuoglu K, Kohli P, Hassabis D. 2021. Highly accurate protein structure prediction with AlphaFold. *Nature* 596:583-589. https://doi.org/10.1038/s41586-021-03819-2.
